# Tudor-SN promotes cardiomyocyte proliferation and neonatal heart regeneration through regulating the phosphorylation of YAP

**DOI:** 10.1186/s12964-024-01715-6

**Published:** 2024-06-28

**Authors:** Chao Su, Jinzheng Ma, Xuyang Yao, Wei Hao, Shihu Gan, Yixiang Gao, Jinlong He, Yuanyuan Ren, Xingjie Gao, Yi Zhu, Jie Yang, Minxin Wei

**Affiliations:** 1 Tianjin Key Laboratory of Cellular and Molecular Immunology, Key Laboratory of Immune Microenvironment and Disease (Ministry of Education), State Key Laboratory of Experimental Hematology, Tianjin Key Laboratory of Metabolic Diseases, The Province and Ministry Co-Sponsored Collaborative Innovation Center for Medical Epigenetics, Tianjin Medical University, Tianjin, China; 2 Division of Cardiovascular Surgery, Cardiac and Vascular Center, the University of Hong Kong-Shenzhen Hospital, Shenzhen, China; 3 Department of Ophthalmology, Tianjin Medical University General Hospital, Tianjin, China

**Keywords:** Tudor-SN, YAP, Cardiomyocyte proliferation, Neonatal heart regeneration

## Abstract

**Background:**

The neonatal mammalian heart exhibits considerable regenerative potential following injury through cardiomyocyte proliferation, whereas mature cardiomyocytes withdraw from the cell cycle and lose regenerative capacities. Therefore, investigating the mechanisms underlying neonatal cardiomyocyte proliferation and regeneration is crucial for unlocking the regenerative potential of adult mammalian heart to repair damage and restore contractile function following myocardial injury.

**Methods:**

The Tudor staphylococcal nuclease (*Tudor-SN*) transgenic (TG) or cardiomyocyte-specific knockout mice (*Myh6-Tudor-SN *^*−/−*^) were generated to investigate the role of Tudor-SN in cardiomyocyte proliferation and heart regeneration following apical resection (AR) surgery. Primary cardiomyocytes isolated from neonatal mice were used to assess the influence of Tudor-SN on cardiomyocyte proliferation in vitro. Affinity purification and mass spectrometry were employed to elucidate the underlying mechanism. H9c2 cells and mouse myocardia with either overexpression or knockout of Tudor-SN were utilized to assess its impact on the phosphorylation of Yes-associated protein (YAP), both in vitro and in vivo.

**Results:**

We previously identified Tudor-SN as a cell cycle regulator that is highly expressed in neonatal mice myocardia but downregulated in adults. Our present study demonstrates that sustained expression of Tudor-SN promotes and prolongs the proliferation of neonatal cardiomyocytes, improves cardiac function, and enhances the ability to repair the left ventricular apex resection in neonatal mice. Consistently, cardiomyocyte-specific knockout of *Tudor-SN* impairs cardiac function and retards recovery after injury. Tudor-SN associates with YAP, which plays important roles in heart development and regeneration, inhibiting phosphorylation at Ser 127 and Ser 397 residues by preventing the association between Large Tumor Suppressor 1 (LATS1) and YAP, correspondingly maintaining stability and promoting nuclear translocation of YAP to enhance the proliferation-related genes transcription.

**Conclusion:**

Tudor-SN regulates the phosphorylation of YAP, consequently enhancing and prolonging neonatal cardiomyocyte proliferation under physiological conditions and promoting neonatal heart regeneration after injury.

**Graphical Abstract:**

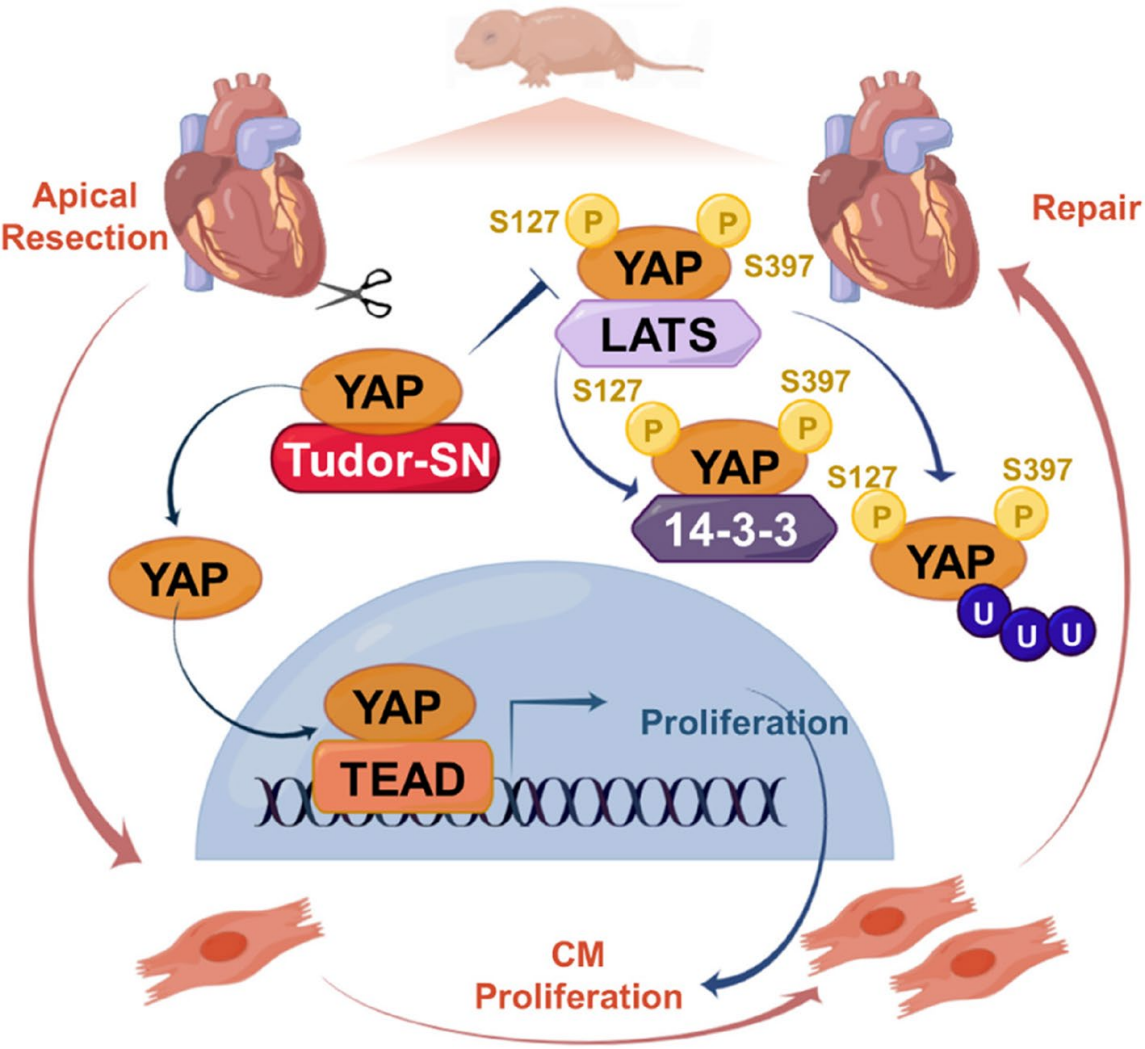

**Supplementary Information:**

The online version contains supplementary material available at 10.1186/s12964-024-01715-6.

## Background

Myocardial infarction or ischemic injury leads to heart failure due to the adult cardiomyocytes have already exited the cell cycle and lost their proliferative ability to repair the damage [1]. Over the past few decades, numerous studies have focused on identifying effective strategies for stimulating the adult heart regeneration. Many studies suggest that heart regeneration can be achieved through pluripotent stem cells differentiation into cardiomyocytes [2, 3] or reprogramming non-cardiomyocytes into cardiomyocytes [4, 5]. Recently, emerging evidences indicate that inducing pre-existing adult cardiomyocyte proliferation may be a novel and effective strategy for promoting heart regeneration after injury [6]. The neonatal heart possesses regenerative capabilities to replenish lost cardiomyocytes through proliferation following injury [7]. Investigations of molecular mechanisms underlying neonatal heart regeneration have provided valuable insights into potential strategies for inducing adult cardiomyocyte re-entry into the cell cycle and restoration of their proliferative ability [8, 9].

The mechanistic target of rapamycin (mTOR) signaling pathway plays a pivotal role in regulating neonatal cardiomyocyte proliferation and embryonic cardiac development [10, 11]. Although mTOR activity is turned off in the adult heart as a protective measure against cardiac aging or hypertrophy [12, 13], recent studies demonstrate that mTOR activation promotes adult cardiomyocyte proliferation and myocardial regeneration during myocardial infarction [14, 15]. The Tudor staphylococcal nuclease (Tudor-SN), also known as staphylococcal nuclease domain containing 1 protein (SND1) or p100, is identified as a novel regulator involved in cell cycle regulation. The expression level of Tudor-SN is closely associated with the cell proliferation capacity that is highly expressed in proliferating cells while significantly reduced in terminally differentiated cells [16]. Recently, we have discovered that the translation efficiency of Tudor-SN is regulated by the mTORC1 pathway. Consistently, Tudor-SN exhibits high expression levels in neonatal myocardia but undergoes dramatic reduction in adult myocardia [17]. In this study, we investigate whether Tudor-SN participates in the regulation of neonatal cardiomyocyte proliferation or regeneration and elucidate its underlying regulatory mechanism.

In this study, we demonstrate that Tudor-SN plays an important role in promoting and prolonging neonatal cardiomyocyte proliferation under physiological conditions, as well as enhancing neonatal heart regeneration following injury. Additionally, we uncover the interaction between Tudor-SN and Yes-associated protein (YAP), which is an integral component of the Hippo-YAP signaling pathway known to be critical for cardiac development and regeneration [18]. The Hippo pathway functions by suppressing cell proliferation through phosphorylation of the downstream effector YAP [19]. Conversely, inhibition of the Hippo pathway or enforced expression of the active YAP has been shown to enhance cardiomyocyte proliferation and increase heart size during embryonic or postnatal stages in mice [20]. Moreover, activation of YAP stimulates adult cardiomyocyte proliferation and facilitates repair after myocardial infarction [21]. Our findings reveal that Tudor-SN prevents large tumor suppressor 1 (LATS1)-mediated phosphorylation of YAP, a key regulator within the Hippo pathway, maintains the stability and promotes the nuclear translocation of YAP, consequently promotes the proliferation- related genes transcription. These results provide valuable insights into the mechanisms underlying neonatal cardiomyocyte proliferation and regeneration while also offering a potential therapeutic target for heart regeneration.

## Materials and methods

### Animals

The C57BL/6 J mice (RRID: 99 IMSR_JAX: 000664) were housed following a 12-h light/dark cycle in SPF facility conditions before and throughout the duration of experiments, with free access to water and the standard laboratory diet, then randomly assigned to the experimental groups. Both male and female mice were included in our study.

The *Tudor-SN* global transgenic (TG) mice were generated by the Model Animal Research Centre of Nanjing Medical University (Nanjing, China) and have been stably bred in our laboratory. The littermates of wild-type (WT) mice were used as control [22]. The gene phenotypes were confirmed through the poly­merase chain reaction (PCR) analysis. Primer sequences can be found in Table S1.

The construction of *Tudor-SN *^*fl/fl*^ mice was previously described in our study [23]. In brief, a *Tudor-SN* clone was isolated from a C57BL/6J mouse genomic DNA library. The targeting vector was designed with two LoxP sites flanking the exon 3 of *Tudor-SN* gene. The *Myh6-Cre* mice (RRID: 011038 from Jackson laboratory) were obtained from Y. Yu lab at Tianjin Medical University. The *Myh6-Cre*-mediated *Tudor-SN* myocardial specific knockout mice (*Myh6-Tudor-SN *^*−/−*^) were generated by crossing *Tudor-SN *^*fl/fl*^ mice with *Myh6-Cre* mice. The littermates were used as negative controls. The gene phenotypes were verified by PCR analysis. Primer sequences are provided in Table S1.

### Cells

The rat cardiomyocyte H9c2 (RRID: CVCL_0286) and HEK293t cell lines (RRID: CVCL_0063) were obtained from the American Type Culture Collection (Manassas, VA). Cells were cultured in Dulbecco’s modified eagle’s medium (DMEM) (Biological Industries, 06-1055-57-1ACS) supplemented with 10% fetal bovine serum (FBS) (Biological Industries, 04-001-1ACS), and incubated at 37ºC with 5% CO_2_.

### Plasmid and lentivirus generation

A stable H9c2 cell line with *Tudor-SN* knockout (*Tudor-SN*-KO) was successfully generated using a modified CRISPR/Cas9 double-nicking gene editing system as previously described [17]. Briefly, two pairs of sgRNAs were designed to specifically target exon 2 of the rat *Tudor-SN* gene. The eukaryotic expression recombinant plasmids PX462-sgRNA-α and PX462-sgRNA-β were constructed using the carrier vector pX462. After the gene sequencing, these two plasmids were co-transfected into H9c2 cells. The primer sequences were shown in the Table S2.

The full-length cDNA sequence of *Tudor-SN* (NM_022694.2) was cloned into the pLVX-IRES-Vector, incorporating a flag tag, to generate the pLVX-IRES-Flag-Tudor-SN plasmid. To construct the pLVX-IRES-Flag-YAP plasmid, the *Yap* cDNA sequence (NM_001394328.1) was cloned into the pLVX-IRES-Vector. The primer sequences can be found in the Table S3.

The HEK293t cells were transfected with the expression plasmid above, which was co-transfected with the packaging plasmid using the polyethyleneimine transfection reagent. The H9c2 WT cells were infected with lentivirus and total protein was extracted to identify infection efficiency.

### Western blot

The cells or mice myocardia were homogenized in the RIPA cell lysis buffer (Beyotime Biotechnology, P0013B) supplemented with phenylmethanesulfonylfluoride (PMSF) and protease inhibitor cocktails (Roche Applied Science, 4693132001). Subsequently, the protein lysate was subjected to SDS-PAGE and transferred onto a membrane for immunoblotting using the specified antibodies. The blots were visualized using chemiluminescence reagents, and the results were quantified using ImageJ (2 ×) software (Rawak Software Inc.). Detailed informations of the antibodies were shown in Table S4.

### Immunohistochemistry

The myocardia of mice were fixed in 4% paraformaldehyde, followed by paraffin embedding and sectioned at the thickness of 7 μm. After deparaffinized and rehydrated, the sections were subjected to antigen retrieval by microwaving for 20 min. The activity of endogenous peroxidase was blocked using peroxide blockers (Origene, PV-9000). Subsequently, the sections were incubated in 5% bovine serum albumin (Genview, FA016) before being incubated with specific antibodies overnight at 4ºC. Sections were then incubated with appropriate secondary antibody before the diaminobenzidine (DAB) staining (Origene, ZLI-9018) on the following day. Images were obtained by microscopy and quantified with ImageJ (2 ×) software. Detailed informations of the antibodies were shown in Table S4.

### Histology

The myocardia tissues were embedded in paraffin prior to obtaining transverse sections of 7 µm thickness encompassing the ventricle. Hematoxylin/eosin (HE) staining and Masson’s trichrome staining were performed following standard procedures [23]. Images were captured using microscopy and quantified with ImageJ (2 ×) software.

### Ratio of heart weight and body weight

To compare the heart weight-to-body weight ratios (HW/BW), the intact hearts of mice at different ages were collected and weighed using an analytical balance after measuring the body weights.

### Immunofluorescence

The myocardial tissues embedded with OCT (SAKURA, 4583) were sectioned at the thickness of 7 μm. The slides were rinsed in PBS prior to blocking with 5% bovine serum albumin, then incubated overnight with specific antibodies. After several washes in PBS, the slides were subsequently incubated with Alexa Fluor 488-or 594-conjugated secondary antibodies (Invitrogen, A21206, 1:1800; Invitrogen, A10036, 1:1800) for 1 h at room temperature. Following this step, the nuclei were fluorescently labeled by staining the slides with DAPI (Sigma, F6057). The fluorescence intensity of the interest regions was acquired using a Leica DMi8 microscope and the LAS X version 3.5.5 software.

The cells were fixed with a 4% paraformaldehyde solution at room temperature for 10 min, followed by permeabilization using 0.3% Triton X-100 (in PBS) and blocking with 5% BSA for 30 min at room temperature. Subsequently, the samples were incubated in buffer containing specific primary antibodies and 1% BSA overnight at 4 °C. After washing with PBS the next day, the cells were incubated with secondary antibodies for 1 h at room temperature before being stained with DAPI for another 5 min. Detailed informations of the antibodies were shown in Table S4.

### Echocardiography

The cardiac function of mice was assessed using transthoracic echocardiography (Visual Sonics Vevo 2000, VSI) as previously described [24]. All the left ventricular measurements were evaluated on anesthetized mice using a mixture of 2% isoflurane (RWD, R510-22-10) and oxygen at a flow rate of 0.5 L/min. The cardiac function was assessed on the long-axis projection with the guide of M-mode following aligning in the transverse B-mode with papillary muscles. The parameters for left ventricular contractile function (ejection fraction, fractional shortening, and the left ventricular posterior wall thickness) were measured or calculated based on the traced anterior and posterior walls of the left ventricle using the electronic caliper in Visual Sonics Vevo Imaging accompanying software.

### Primary cells isolation and culture

Primary cardiomyocytes and fibroblasts were isolated from neonatal mice at P1 or P7. The neonatal mice were first washed with 75% alcohol before the hearts were surgically removed and placed in pre-cooled PBS. The hearts were then cut into pieces (~ 1 mm^3^) using surgical scissors. Subsequently, the tissues were dissociated in Hank’s Balanced Salt Solution (Beyotime, C0219) containing 0.125 mg/mL trypsin (Gibco, 15090046), 0.1 mg/mL collagenase type II (Solarbio, C8150) and 10 mg/mL DNase I (Solarbio, D5220). The supernatants were centrifuged at 4 °C at 1000 rpm/min for 10 min, the sediment was resuspended in DMEM supplemented with 20% FBS and filtered by 70 μm filter. The resuspended cells were planted in culture dish for 2 h to separate fibroblast, after which the cardiomyocytes in the supernatant were collected and seeded into a new dish.

### Flow cytometry

Total cell suspensions from the heart of WT or TG mice were prepared as described above. The resulting cell suspensions were filtered through 70 μm filters (BIOLOGIX, 15-1040) before erythrocytes were lysed using erythrocyte lysis buffer (Solarbio, R1010). After determining the total cell count by using the Countess™ II FL automated cell counter (Invitrogen, C10227), single cell suspension was divided equally into three parts. The cells were washed in PBS for 3 times before fixed and permeabilized using Fixation Buffer (Biolegend, 420801) and Intracellular Staining Permeabilization Wash Buffer (Biolegend, 421002) according to the manufacturer's instructions. To identify cardiomyocytes (CMs), cardiac fibroblast (FBs) and endothelial cells (ECs), cell suspensions were stained with anti-TNNT2, anti-Vimentin (VIM) and anti-CD31 antibodies respectively for 1 h at 4 °C. Cells were then washed and incubated with Alexa Fluor 488-conjugated secondary antibodies (Invitrogen, A21206, 1:1000) for 1 h at room temperature. After washing with PBS, the cells were sorted by flow cytometry on a FACS Aria Flow Cytometer (BD Biosciences FACSVerse, Becton Dickinson) and the percentage of positive cells were determined. Cardiomyocytes were identified as TNNT2^+^, fibroblasts as VIM^+^, and endothelial cells as CD31^+^ cells. The number of each specific cell type equals the total number of cells multiplied by the percentage of each specific cell. FlowJo V10 software (version 10.4) was used to process flow cytometry data. The informations of antibodies were shown in Table S4.

### Neonatal mouse heart apical resection model

Neonatal mouse heart apical resection (AR) was performed as previously described [25]. Briefly, neonates were anesthetized on an ice bed for 4 min. Following skin incision, a lateral thoracotomy at the fourth intercostal space was performed by blunt dissection of the intercostal muscles. Hearts were carefully exteriorized from the chest by using steady pressure on the abdomen, and then 15-20% of the ventricular apex was resected using iridectomy scissors. Following apical resection, the thoracic wall incision was closed with 7.0 non-absorbable silk sutures and skin adhesive (Vetbond Tissue Adhesive, 1469SB). Sham-operated mice underwent the same procedure without apical resection. The neonatal mice were then placed on a warm platform at 37 °C until fully recovery.

### RNA extraction and quantitative real-time PCR (qRT-PCR)

The TRIzol reagent (Vazyme, R401-01) was utilized for total RNA extraction from the cells or mouse myocardia following the manufacture’s protocol. The cDNA synthesis was performed using the Revert Aid First Strand cDNA Synthesis Kit (Thermo Fisher Scientific, K1622) according to the manufacturer's instructions. PCR analysis was carried out using SYBR Green (Vazyme Biotech, Q131-02) on a Step One Real-Time PCR System (Thermo Fisher Scientific, ABI-StepOne Plus). α-Tubulin served as an endogenous control for normalization purposes. All data were analyzed by the 2^−ΔΔCT^ method. The primer sequences can be found in the Table S5.

### Wheat germ agglutinin staining

To analyse the size of cardiomyocyte, mice myocardia were embedded using paraffin and sectioned, followed by staining with fluorescein-conjugated wheat germ agglutinin (WGA, Invitrogen, W7024). The average size of cardiomyocyte was determined by measuring the cross-sectional area of individual cells and quantified with ImageJ (2 ×) software.

### TUNEL assay

Apoptosis in mice cardiac tissue was detected by TUNEL detection kit (Beyotime Biotechnology, C1091) following the manufacturer’s instructions. Briefly, paraffin sections were deparaffinized, rehydrated and underwent antigen retrieval before being incubated with the TUNEL reaction mixture. After DAB staining, nuclei were visualized with hematoxylin staining. Images were captured using microscope Leica DMi8 and the software LAS X version 3.5.5.

### Silver staining and mass spectrometry assay

The silver staining and mass spectrometry assay were conducted as previously described [26]. H9c2 cells were infected with pLVX-IRES-Flag-Tudor-SN plasmids or pLVX-IRES-Flag-vector. Cell lysates were subjected to immunoprecipitation using anti-Flag agarose beads (Abcam, ab270704) followed by eluting with Flag peptide (Thermo Fisher Scientific, PEP-087). The protein was visualized on 8% SDS-PAGE by silver staining using the Pierce silver stain kit (Thermo Fisher Scientific, 24612). Distinct protein bands were excised and subjected to in-gel tryptic digestion. The resulting peptides were separated using reverse-phase liquid chromatography on an easy-nLC 1000 system (Thermo Fisher Scientific) and directly sprayed into a Q Exactive Plus mass spectrometer (Thermo Fisher Scientific). Mass spectrometry analysis was carried out in data-dependent mode with automatic switch between a full MS and an MS/MS scan in orbitrap high resolution mass spectrometry. The 10 most intense peaks with charge states ≥ 2 were selected for fragmentation by higher-energy collision dissociation with a normalized collision energy of 27%. MS2 spectra were acquired at a resolution of 17500. The exclusion window was set to ± 2.2 Da. All MS/MS spectra were searched against the Uniport-Mice protein sequence database by using the PD search engine (v 2.1.0, Thermo Fisher Scientific) with an overall false discovery rate (FDR) for peptides of less than 1%.

### Co-immunoprecipitation assay

The cellular lysates were prepared using 1% NP-40 lysis buffer [0.2% Nonidet P­40, 150 mM NaCl, 2 mM EDTA and 50 mM tris­l-HCl (pH = 8.0)], supplemented with PMSF and protease inhibitor cocktails. For immunoprecipitation, specific antibodies were incubated with Pierce Protein A/G agarose (Thermo Pierce, 20422) at 4 °C for 6 h. Ten percent of the total protein lysates were used as input for each sample. After washing, the supernatant was discarded and the proteins were eluted from the beads by resuspending them in loading buffer. The precipitated proteins were subsequently analyzed using SDS-PAGE and western blot techniques. The informations of antibodies were shown in Table S4.

### Recombinant protein purification and GST-pulldown

The glutathione S-transferase (GST)-Tudor-SN, GST-YAP, as well as the truncations GST plasmids were expressed in *Escherichia coli* (*E. coli*) following by adding 0.2 mM isopropyl β-D-1-thiogalactopyranoside (IPTG) (Beyotime, ST098). After sonication-induced lysis on ice and removal of precipitation, the lysate was incubated with glutathione-agarose beads for 10 h. Recombinant protein expression was carried out using rabbit reticulocyte lysate (TNT systems) (Promega, L1171), following the manufacturer's recommended protocol. The GST fusion proteins which bounded with beads were incubated with transcribed/translated products described above for an additional 12 h in vitro. The precipitated proteins were analyzed by SDS-PAGE and western blot analysis. The primer sequences are shown in the Table S6.

### Isolation of nuclear and cytoplasm

The cells and mice myocardia were isolated in cytoplasmic lysis buffer [1% Nonidet P­40, 1.5 mM MgCl_2_, 10 mM KCl, and 10 mM tris­-HCl (pH = 8.0)]. After centrifugation, the precipitation was separated as nuclear while the supernatant as cytoplasmic fraction. Following the separation of supernatant and precipitation, nuclear lysis buffer [0.2 mM EDTA, 1 mM DTT, 400 mM NaCl, 5% Glycerol, 20 mM tris­-HCl (pH = 8.0), and 1.5 mM MgCl_2_] was added to the precipitate fraction for the lysis of nuclear.

### Data and statistical analysis

The statistical analysis was conducted with GraphPad Prism software (GraphPad Software Inc., version 7.00). Data from triplicate biological experiments are presented as the mean ± standard error of mean (SEM). The two-tailed independent samples t-test was employed to compare the data from two distinct groups. The one way analysis of variance (ANOVA) was used to compare multiple groups of data. The repeated measures ANOVA was used to compare different time changes data. The ANOVA was followed by Bonferroni multiple-comparison post-hoc correction. Statistical significance was set at *P* < 0.05.

## Results

### The neonatal cardiomyocytes of *Tudor-SN* transgenic mice exhibit enhanced proliferation and regeneration ability

**Fig. 1:**
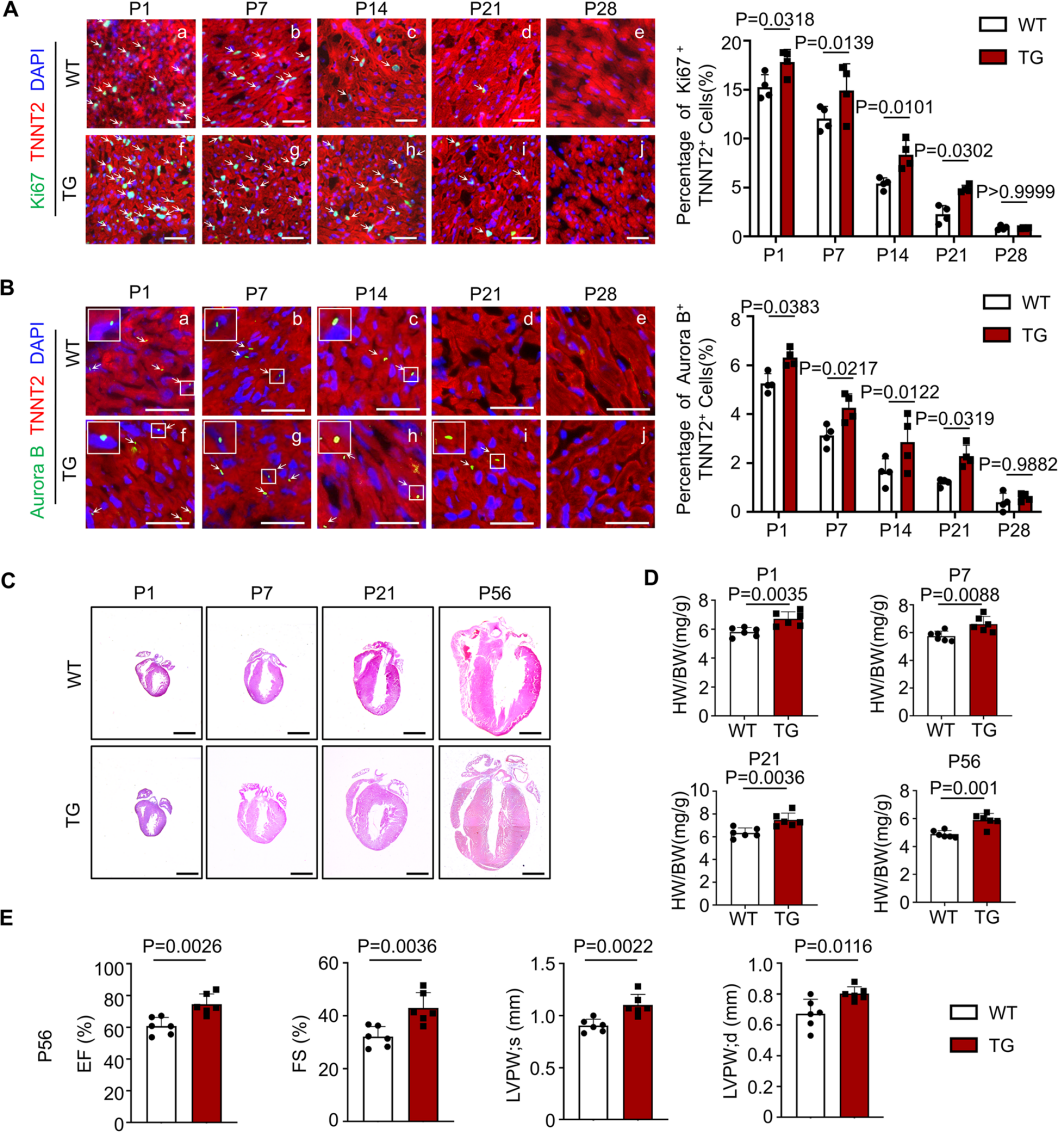
Overexpression of Tudor-SN protein promotes neonatal cardiomyocyte proliferation and improves cardiac function. Myocardia tissues from postnatal day 1 (P1), P7, P14, P21 and P28 WT and TG mice were sliced and subjected to immunofluorescence staining with an anti-cardiac troponin T (TNNT2, red) antibody to mark cardiomyocytes, while DAPI (blue) as a nuclear counter stain. **A** Immunofluorescence staining with an anti-Ki67 (green) antibody was used to mark cell proliferation. Representative immunostaining and percentage of Ki67^+^ TNNT2^+^ cells were shown (Scale bar, 50 μm; n = 4 biological replicates). **B** Immunofluorescence staining with an anti-Aurora B (green) antibody was used to mark cytokinesis. Representative immunostaining and percentage of Aurora B^+^ TNNT2^+^ cells were shown (Scale bar, 50 μm; n = 4 biological replicates). **C** Representative HE staining of hearts from P1, P7, P21, and P56 WT and TG mice (Scale bar, 1 mm). **D** The heart-to-body weight ratio (HW/BW) of WT and TG mice at P1, P7, P21 and P56 (n = 6 biological replicates). **E** Echocardiography was used to assess the cardiac function of WT and TG mice at P56 (n = 6 biological replicates). EF, ejection fraction; FS, fractional shortening; LVPW, left ventricular posterior wall. All data were presented as the mean ± SEM, results in A-B were analyzed by repeated measures ANOVA followed by Bonferroni post-hoc correction, and unpaired two-tailed Student’s t-test were used in D-E

Tudor-SN, a regulator in the cell cycle and, is capable of promoting cell proliferation [16]. Recently, we reported that Tudor-SN is involved in mTORC1-mediated regulation of H9c2 cell proliferation [17]. To further elucidate the potential function of Tudor-SN in cardiomyocyte proliferation, we utilized global *Tudor-SN* transgenic (TG) mice (Fig. S1A) to assess the proliferative ability of cardiomyocyte at different ages compared to wild-type (WT) mice. Western blot (WB) and immunohistochemical staining (IHC) demonstrated that the expression of Tudor-SN in heart tissues progressively declined with age in WT mice but remained consistently high in TG mice across all ages (Fig. S1B-C). Proliferation marker Ki67, cytokinesis marker Aurora B, and mitosis marker phosphorylated histone 3 (p-H3) were employed to evaluate the proliferative ability of cardiomyocytes. In general, immunostaining revealed that cardiomyocytes from WT mice mostly exited cell cycle at P14 (Fig. 1A, c; B, c; Fig. S2A, c), which was consistent with previous report [27]. Meanwhile, we observed that highly expressed Tudor-SN promoted cardiomyocyte proliferation at P1 and P7 in TG mice as evidenced by an increase in the proportion of Ki67^+^ TNNT2^+^ cells (Fig. 1A, f, g), Aurora B^+^ TNNT2^+^ cells (Fig. 1B, f, g), and p-H3^+^ TNNT2^+^ cells (Fig. S2A, f, g), and extended the proliferative window from P14 to P21 (Fig. 1A, i; B, i; Fig. S2A, i). Additionally, Ki67 staining and EdU incorporation assay revealed an increase in the proportion of Ki67^+^ TNNT2^+^ and EdU^+^ TNNT2^+^ primary cardiomyocytes isolated from in TG mice compared to WT mice at P1 (Fig.S2B, C). Moreover, an increase in the percentage of mononucleated primary cardiomyocytes (Fig. S2D) and a higher total number of cardiomyocytes were observed in the P7 TG mice (Fig. S4A). However, there were no significant differences of cardiomyocyte proliferation ability between TG (Fig. 1A, j; B, j; Fig. S2A, j) and WT (Fig. 1A, e; B, e; Fig. S2A, e) mice at P28. These findings illustrate that Tudor-SN is likely to promote and prolong postnatal cardiomyocyte proliferation but does not have the capacity to retain the cardiomyocyte cell cycle.

The hematoxylin–eosin staining (HE) of cardiac morphology (Fig. 1C) and the heart-to-body weight ratio (HW/BW) (Fig. 1D) demonstrated that TG mice at P1, P7, P21, and P56, had relatively “larger” hearts compared to WT mice. Furthermore, the results showed no differences in the size of cardiomyocytes between WT and TG mice (Fig. S3A), as well as the mRNA levels of hypertrophy markers natriuretic peptide A (*Nppa*) and natriuretic peptide B (*Nppb*) (Fig. S3B). Meanwhile, no cardiac fibrosis was observed in TG mouse hearts through Masson staining (Fig. S3C), and Tudor-SN overexpression did not affect the proliferation ability of cardiac fibroblasts and endothelial cells (Fig. S3D-F; Fig. S4, B, C). These findings demonstrate that the “larger” heart in TG mice was not caused by pathological hypertrophy or non-cardiomyocytes proliferation, but rather due to enhanced neonatal cardiomyocyte proliferation.

We subsequently performed echocardiography to explore the impact of Tudor-SN on cardiac function. As shown in Fig. 1E, adult TG mice exhibited elevated ejection fraction (EF) and fractional shortening (FS), indicative of enhanced cardiac function. Moreover, echocardiography revealed increased thickness of the left ventricular posterior wall (LVPW) in TG mice compared to WT mice during both systolic and diastolic state. These findings collectively demonstrate that Tudor-SN promotes and prolongs neonatal cardiomyocyte proliferation, consequently enhancing cardiac function under physiological condition.

**Fig. 2:**
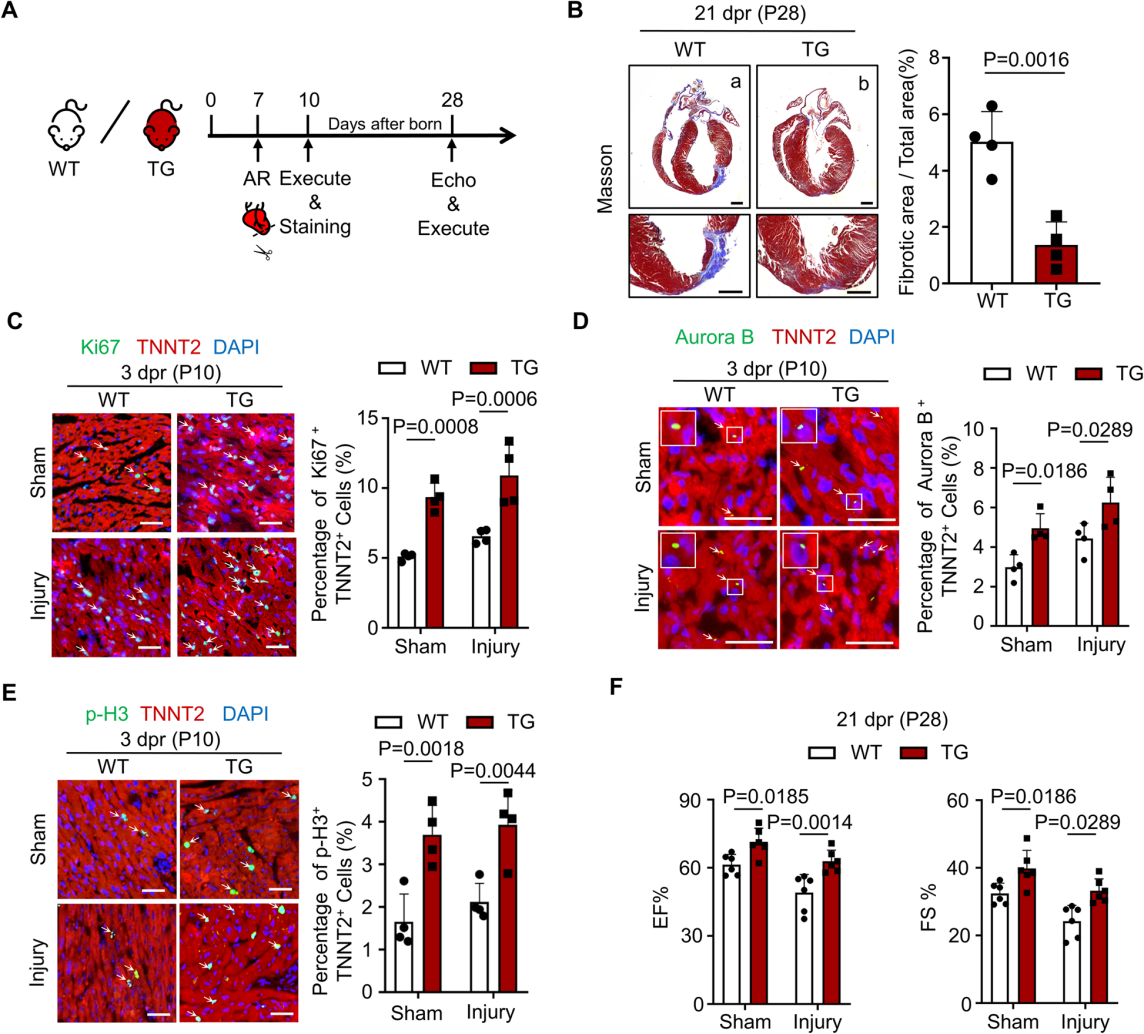
Tudor-SN protein promotes neonatal heart regeneration after AR injury. AR injury models were generated in WT and TG neonatal mouse at P7. Myocardia tissues were collected at 3 dpr (P10) and 21 dpr (P28). **A** Experimental timeline. AR, apical resection; Echo, echocardiography. **B** Masson’s trichrome staining showed heart regeneration in WT and TG mice at 21 dpr (P28), with statistical analysis of the left ventricular scar size quantified by the fibrotic area relative to the myocardial area (Scale bar, 1 mm; n = 4 biological replicates). **C**-**E** The myocardia tissue sections at 3 dpr (P10) were stained with anti-Ki67 (green) (**C**), anti-Aurora B (green) (**D**), or anti-p-H3 (green) (**E**) antibodies to demonstrate the proliferation, cytokinesis or mitosis of cells. TNNT2 (red) marked cardiomyocytes, while DAPI (blue) stained nuclei (Scale bar, 50 μm). The percentage of Ki67^+^ TNNT2^+^, Aurora B^+^ TNNT2^+^, and p-H3^+^ TNNT2^+^ cardiomyocytes per section were quantified (n = 4 biological replicates) (**F**) Cardiac function of WT and TG mice at 21dpr (P28) were detected by echocardiography (n = 6 biological replicates). All data were presented as the mean ± SEM, results in B were analyzed by unpaired two-tailed Student’s t-test, results in C-F were analyzed by one-way ANOVA follow by Bonferroni post-hoc correction

To investigate whether Tudor-SN is involved in neonatal heart regeneration following injury, we performed apical resection (AR) surgery on neonatal mice. Previous studies have revealed that P1 WT mice can fully regenerate their myocardium at 21-day post resection (dpr), whereas P7 mice cannot [25, 27]. In order to better elucidate the influence of Tudor-SN on heart regeneration, we performed AR operation on both TG and WT neonatal mice at P7 (Fig. 2A). Masson staining showed nearly complete myocardial regeneration in P7 TG mice at 21 dpr (P28) (Fig. 2B, b), while P7 WT mice heart failed to fully recover (Fig. 2B, a), which is consistent with previous studies [27]. Additionally, immunostaining demonstrated an increased proportion of Ki67^+^ TNNT2^+^, Aurora B^+^ TNNT2^+^, and p-H3^+^ TNNT2^+^ cardiomyocytes in TG mice compared to WT mice at 3 dpr (P10) (Fig. 2C-E). Furthermore, evidence from echocardiography indicated that Tudor-SN promoted functional recovery in TG mouse hearts at 21 dpr (P28) (Fig. 2F). These data demonstrate that overexpression of Tudor-SN enhances neonatal cardiomyocyte proliferation and facilitates heart regeneration following cardiac injury.

### The proliferation and regeneration abilities of neonatal cardiomyocytes are hindered by the cardiomyocyte-specific knockout of *Tudor-SN*

**Fig. 3:**
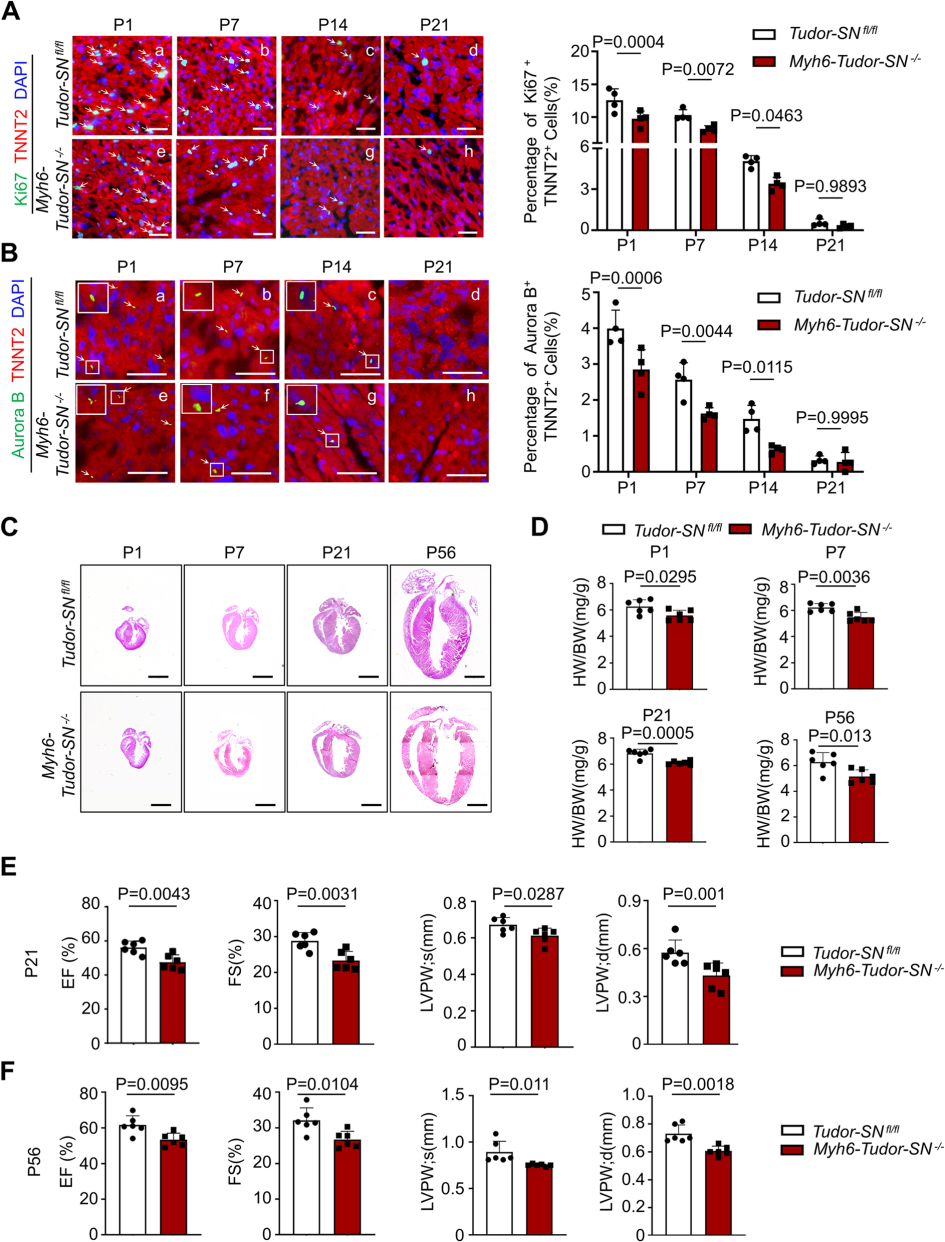
Knockout of *Tudor-SN* inhibits neonatal cardiomyocyte proliferation and weakens cardiac function of mice. Myocardia tissues from P1, P7, P14 and P21 *Tudor-SN *^*fl/fl*^ and *Myh6-Tudor-SN *^*−/−*^ mice were sliced and subjected to immunofluorescence staining with an anti- TNNT2 (red) antibody to mark cardiomyocytes, while DAPI (blue) to mark nucleus. **A** Representative immunostaining and quantitative analysis of Ki67^+^ TNNT2^+^ cells were shown (Scale bar, 50 μm; n = 4 biological replicates). **B** Representative immunostaining and quantitative analysis of Aurora B^+^ TNNT2^+^ cells were shown (Scale bar, 50 μm; n = 4 biological replicates). **C** Representative HE staining of hearts from P1, P7, P21 and P56 *Tudor-SN *^*fl/fl*^ and *Myh6-Tudor-SN *^*−/−*^ mice (Scale bar, 1 mm). **D** The HW/BW ratio of *Tudor-SN *^*fl/fl*^ and *Myh6-Tudor-SN *^*−/−*^ mice at P1, P7, P21 and P56 (n = 6 biological replicates). **E**, **F** Cardiac function of *Tudor-SN *^*fl/fl*^ and *Myh6-Tudor-SN *^*−/−*^ mice at P21 and P56 were detected by echocardiography (n = 6 biological replicates). All data were presented as the mean ± SEM, results in A, B were analyzed by repeated measures ANOVA followed by Bonferroni post-hoc correction, unpaired two-tailed Student’s t-test were used in C-F

To further confirm the function of Tudor-SN in cardiomyocyte proliferation, we generated cardiomyocyte-specific *Tudor-SN* knockout mice (*Myh6-Tudor-SN *^*−/−*^) (Fig. S5). Immunostaining analysis revealed that the cardiomyocytes of *Tudor-SN *^*fl/fl*^ mice gradually lost the ability of cell proliferation at P7 (Fig. 3A, b; B, b; Fig. S6A, b) and exited cell cycle at P14 (Fig. 3A, c; B, c; Fig. S6A, c), which was consistent with the WT mice (Fig. 1A-B; Fig. S2A). The knockout of *Tudor-SN* reduced the proportion of Ki67^+^ TNNT2^+^ cells (Fig. 3A, e–g), Aurora B^+^ TNNT2^+^ cells (Fig. 3B, e–g), and p-H3^+^ TNNT2^+^ cells (Fig. S6A, e–g) in *Myh6-Tudor-SN *^*−/−*^ mice compared to *Tudor-SN *^*fl/fl*^ mice at P1, P7, and P14. Furthermore, our results demonstrated that *Tudor-SN* knockout inhibited the primary cardiomyocyte proliferation as evidenced by a decrease of Ki67^+^ TNNT2^+^ cells, EdU^+^ TNNT2^+^ cells, and percentage of mononucleated primary cardiomyocytes in the *Myh6-Tudor-SN *^*−/−*^ mice (Fig. S6B-D). These findings support the opinion that the absence of Tudor-SN in neonatal mouse cardiomyocytes resulted in a reduction in cardiomyocyte proliferation. Accordingly, thinner left ventricle wall and reduced HW/BW ratio were observed in the *Myh6-Tudor-SN *^*−/−*^ mice compared to *Tudor-SN *^*fl/fl*^ mice at P1, P7, P21, and P56 (Fig. 3C, D). Moreover, echocardiography revealed impaired cardiac function and thinner left ventricle wall in *Myh6-Tudor-SN *^*−/−*^ mice at P21 and P56 (Fig. 3E, F). We also observed that the cardiac fibrosis, cardiomyocyte size and apoptosis were not affected by the knockout of *Tudor-SN* (Fig. S7). These results indicate that *Tudor-SN* knockout reduced heart function through inhibiting the CM proliferation.

**Fig. 4:**
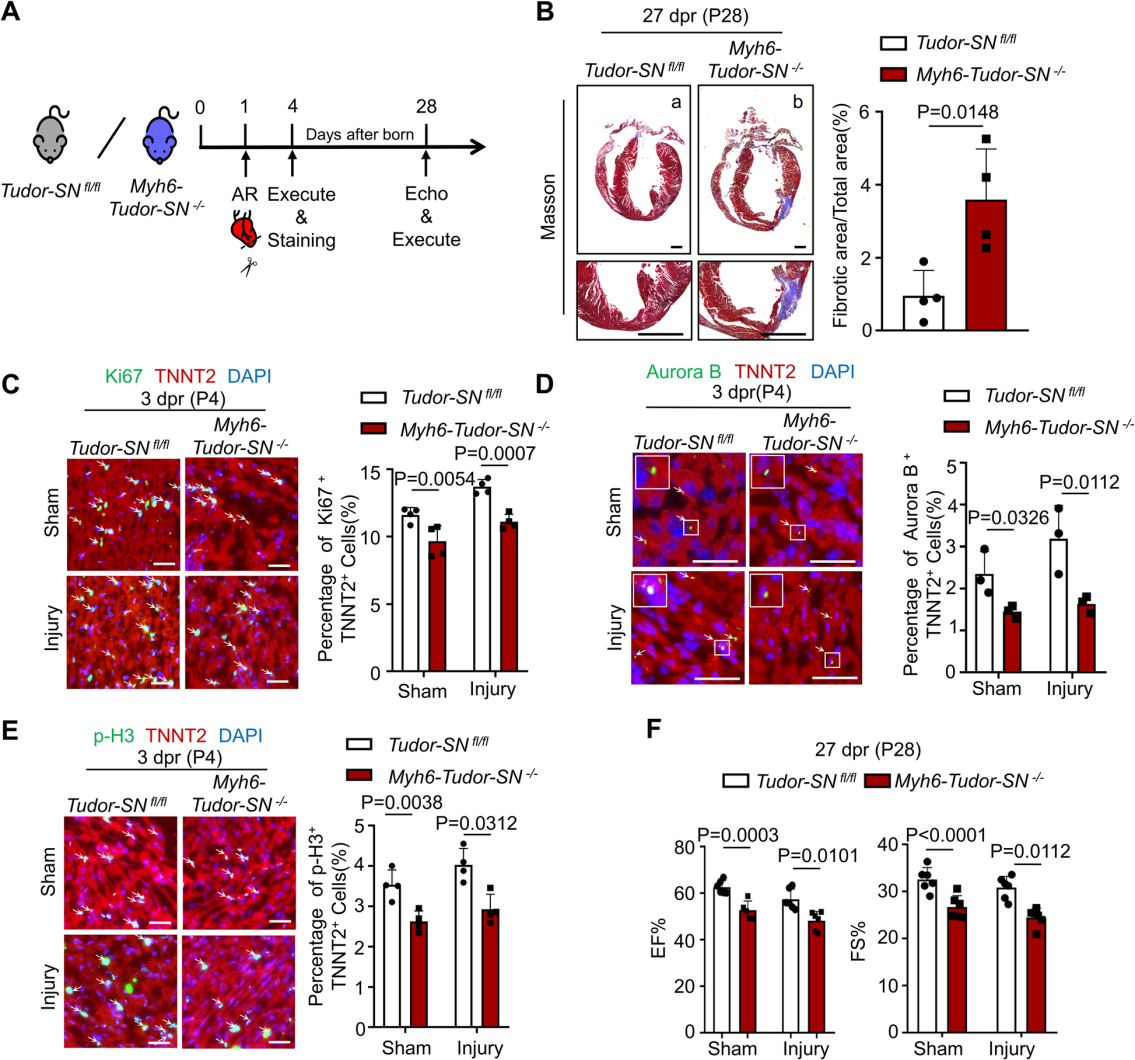
Knockout of *Tudor-SN* retards the neonatal heart regeneration. AR injury models were generated in *Tudor-SN *^*fl/fl*^ and *Myh6-Tudor-SN *^*−/−*^ neonatal mouse at P1. The myocardia tissues were collected at 3 dpr (P4) and 27 dpr (P28). **A** Experimental timeline. **B** Masson’s trichrome staining showed heart regeneration in *Myh6-Tudor-SN *^*−/−*^ mice and *Tudor-SN *^*fl/fl*^ littermates at 27 dpr (P28) (Scale bar, 1 mm), with statistical analysis of the left ventricular scar size quantified by the fibrotic area relative to the myocardial area in trichrome-stained sections (n = 4 biological replicates). **C**-**E** The myocardia tissue sections at 3 dpr (P4) were stained with anti-Ki67 (green) (**C**), anti-Aurora B (green) (**D**), or anti-p-H3 (green) (**E**) antibodies to demonstrate the proliferation, cytokinesis or mitosis of cells. TNNT2 (red) was used to mark cardiomyocytes, while DAPI (blue) was used to stain nuclei (Scale bar, 50 μm). Percentage of Ki67^+^ TNNT2^+^, Aurora B^+^ TNNT2^+^, and p-H3^+^ TNNT2^+^ cardiomyocytes per section were shown (n = 4 biological replicates in C, E; *n* = 3 biological replicates in D). **F** Cardiac function of *Tudor-SN *^*fl/fl*^ and *Myh6-Tudor-SN *^*−/−*^ mice were detected at 27 dpr (P28) by echocardiography (n = 6 biological replicates). All data were presented as the mean ± SEM, results in B were analyzed by unpaired two-tailed Student’s t-test, results in C-F were analyzed by one-way ANOVA follow by Bonferroni post-hoc correction

The AR surgery was also conducted in the P1 *Myh6-Tudor-SN *^*−/−*^ and *Tudor-SN *^*fl/fl*^ mice to assess the influence of *Tudor-SN* knockout on heart regeneration (Fig. 4A). As shown in Fig. 4B, *Tudor-SN *^*fl/fl*^ mice almost regenerated myocardium at 27 dpr (P28), whereas knockout of *Tudor-SN* retarded the myocardium repair in *Myh6-Tudor-SN *^*−/−*^ mice following injury. Furthermore, the proportions of Ki67^+^ TNNT2^+^, Aurora B^+^ TNNT2^+^, and p-H3^+^ TNNT2^+^ cells were significantly reduced in *Myh6-Tudor-SN *^*−/−*^ mice at 3 dpr (P4) compared to *Tudor-SN *^*fl/fl*^ mice (Fig. 4C-E), and functional recovery was impeded in *Myh6-Tudor-SN *^*−/−*^ mice at 27 dpr (P28) (Fig. 4F). Collectively, these findings demonstrate that Tudor-SN plays a vital role in neonatal cardiomyocyte proliferation and heart regeneration following cardiac injury.

### Tudor-SN interacts with YAP and retards the interaction between YAP and LATS1

**Fig. 5:**
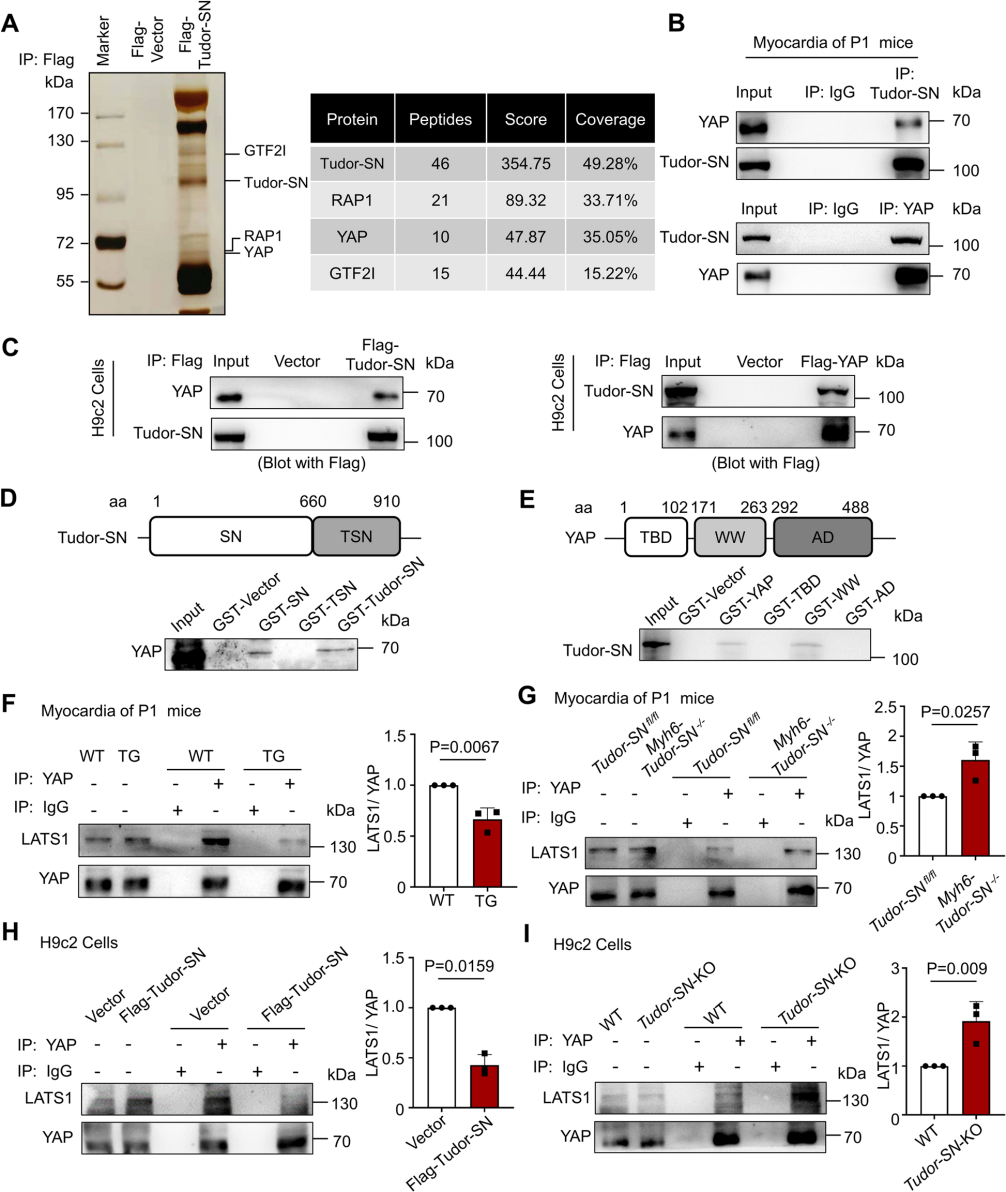
Tudor-SN prevents LATS1 to interact with the WW domain of YAP. **A** (Left) Lysates of H9c2 cells transfected with pLVX-IRES-Flag-Tudor-SN (Flag-Tudor-SN) plasmids or pLVX-IRES-Flag-vector (Flag-Vector) were purified with anti-Flag beads and eluted with SDS buffer. The eluates were subjected to SDS-PAGE and silver staining. (Right) Mass spectrometry identified several interacting proteins of Tudor-SN. Peptide coverage of indicated proteins was shown. **B** Lysates of P1 WT mice myocardial were immunoprecipitated with anti-Tudor-SN or anti-YAP antibodies respectively, western blot detected the interaction of Tudor-SN and YAP protein. **C** Total lysates of H9c2 cells with Flag-Tudor-SN or Flag-YAP expression was immunoprecipitated with anti-Flag beads, followed by western blot with antibodies against the YAP or Tudor-SN. **D** GST pull-down analysis of the interaction of GST-fusion protein containing full-length Tudor-SN (GST-Tudor-SN), SN domain (GST-SN), and TSN domain (GST-TSN) with in vitro-translated YAP from rabbit reticulocytes, followed by western blot with antibodies against the YAP. **E** GST pull-down analysis of the interaction of GST-fusion protein full-length YAP (GST-YAP), TBD domain (GST-TBD), WW domain (GST-WW), and AD domain (GST-AD) with in vitro-translated Tudor-SN from rabbit reticulocytes, followed by western blot with antibodies against the Tudor-SN. **F**, **G** Total lysates of P1 WT and TG mice myocardial, or myocardial of *Tudor-SN *^*fl/fl*^ and *Myh6-Tudor-SN *^*−/−*^ mice were immunoprecipitated with anti-YAP antibodies, western blot detected the interaction of YAP protein and LATS1. **H**, **I** Total lysates of WT and *Tudor-SN*-KO H9c2 cells, or total lysates of H9c2 cells transfected with Flag-Vector or Flag-Tudor-SN were immunoprecipitated with anti-YAP antibodies, western blot detected the interaction of YAP protein and LATS1. The western blot results were analyzed by ImageJ (2 ×) software (n = 3 biological replicates). All data were presented as the mean ± SEM, unpaired two-tailed Student’s t-test were used

To elucidate the underlying mechanisms of Tudor-SN protein regulated cardiomyocyte proliferation, we employed affinity purification and mass spectrometry to identify the proteins associated with Tudor-SN in H9c2 cells expressing Flag-Tudor-SN proteins stably. The result revealed that Tudor-SN co-purified with multiple cell proliferation-related proteins, including YAP protein (Fig. 5A). YAP, functioning as a transcriptional co-activator, plays important roles in promoting cardiomyocyte proliferation mainly through binding with transcription factors [28]. Therefore, we first detected the physical interaction between Tudor-SN and YAP in myocardia of P1 WT mice by Co-immunoprecipitation assay (Co-IP). As shown in Fig. 5B, the endogenous YAP was precipitated with anti-Tudor-SN antibody, vice versa, Tudor-SN was precipitated with anti-YAP antibody. Furthermore, the physical interaction between Tudor-SN and YAP was validated by ectopically overexpression of Flag-Tudor-SN or Flag-YAP in H9c2 cells and immunoprecipitation with anti-Flag immunomagnetic beads (Fig. 5C). Meanwhile, to map the interaction domain between Tudor-SN and YAP, we employed glutathione S-transferase (GST) pull-down assay. As shown in Fig. 5D, both full-length Tudor-SN or SN domain efficiently associated with YAP while TSN domain did not exhibit such association, while full-length YAP or WW domain efficiently associated with Tudor-SN (Fig. 5E). The GST-fusion proteins were visualized through Coomassie blue staining (Fig. S8).

The binding of YAP to LATS1, the core component of the mammalian Hippo pathway, through its WW domain leads to subsequent phosphorylation [29]. Our results show that Tudor-SN also interacts with the WW domain of YAP, suggesting a potential role for Tudor-SN in hindering the interaction between LATS1 and YAP. To verify our hypothesis, we performed Co-IP experiments to examine the physical interaction between LATS1 and YAP in myocardia of WT and TG mice at P1. As shown in Fig. 5F, overexpression of Tudor-SN weakened the interaction between YAP and LATS1 in myocardia of P1 TG mice. Conversely, knockout of *Tudor-SN* enhanced their binding in myocardia of P1 *Myh6-Tudor-SN *^*−/−*^ mice (Fig. 5G). Additionally, Tudor-SN protein was ectopically overexpressed in the H9c2 cells through infecting with pLVX-IRES-Flag-Tudor-SN plasmids (Flag-Tudor-SN) or knocked out by the modified CRISPR/Cas9 system (*Tudor-SN* -KO), followed by detection of interactions between YAP and LATS1. As shown in Fig. 5H-I, similar results were obtained from H9c2 cells, that overexpression of Tudor-SN attenuated the interaction between YAP and LATS1, whereas knockout of *Tudor-SN* enhanced their binding.

### Tudor-SN inhibits the phosphorylation of YAP and enhances its stability

**Fig. 6:**
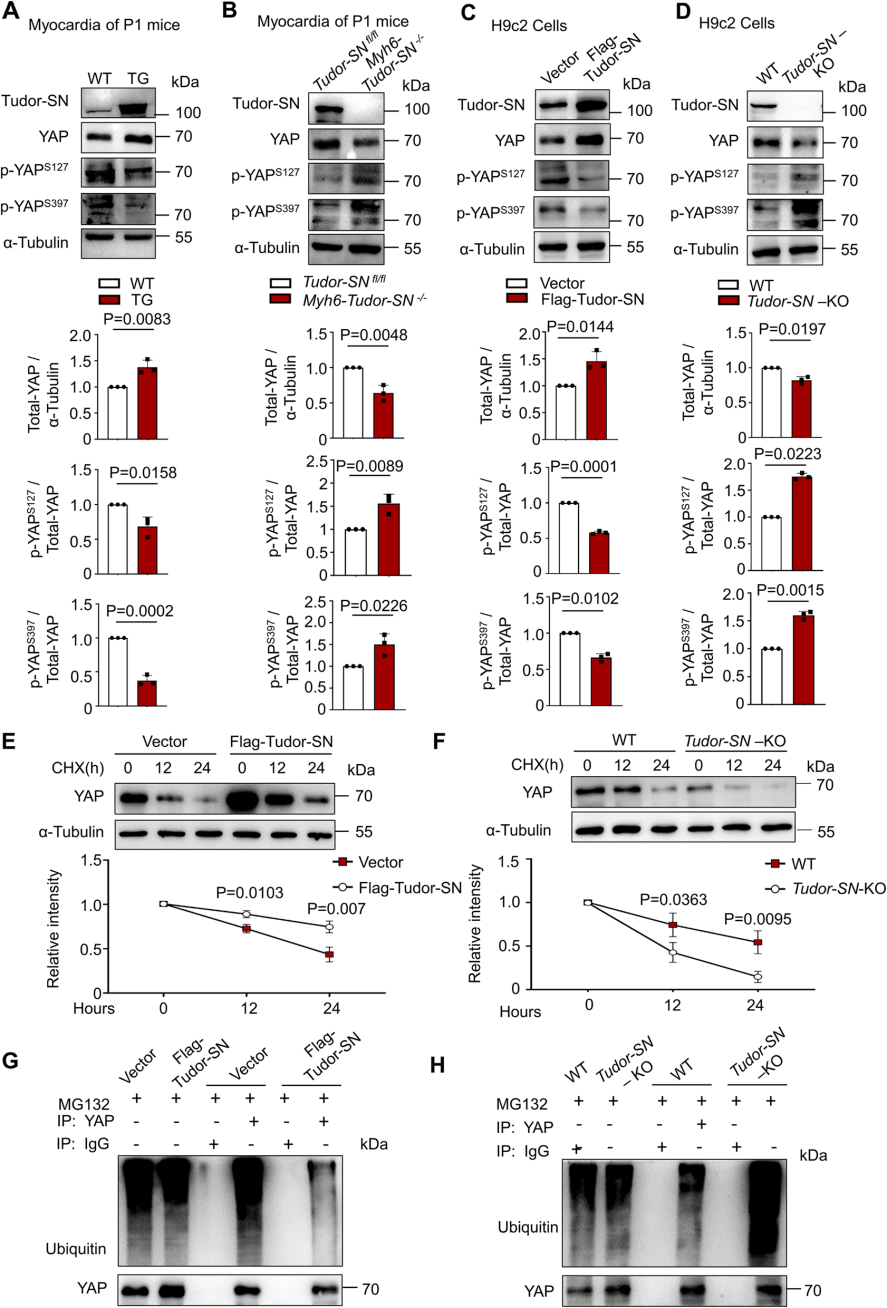
Tudor-SN inhibits the phosphorylation and maintains the stability of YAP. **A**, **B** The expression levels of Tudor-SN, YAP, p-YAP^S127^ and p-YAP^S397^ in myocardia of WT and TG mice, as well as myocardia of *Tudor-SN *^*fl/fl*^ and *Myh6-Tudor-SN *^*−/−*^ mice at P1 were detected by western blot (n = 3 biological replicates). **C**, **D** The expression levels of Tudor-SN, YAP, p-YAP^S127^ and p-YAP^S397^ in H9c2 cells transfected with Flag-vector or Flag-Tudor-SN, or in WT and *Tudor-SN*-KO H9c2 cells were detected by western blot (n = 3 biological replicates). **E**, **F** The effect of Tudor-SN on the half-life of YAP was evaluated in H9c2 cells transfected with Flag-vector or Flag-Tudor-SN, as well as WT or *Tudor-SN-*KO H9c2 cells treated with CHX (50 μg/ml) and harvested at 0, 12, 24 h. The expression level of YAP was detected by western blot (n = 3 biological replicates). **G**, **H** Total lysates of H9c2 cells transfected with Flag-vector or Flag-Tudor-SN, as well as total lysates of WT and *Tudor-SN*-KO H9c2 cells were immunoprecipitated with anti-YAP antibodies, western blot detected the ubiquitination of YAP. The western blot results were analyzed by ImageJ (2 ×) software. All data were presented as the mean ± SEM, unpaired two-tailed Student’s t-test were used in A-D, results in E-F were analyzed by repeated measures ANOVA followed by Bonferroni post-hoc correction

LATS1 directly phosphorylate YAP at Serine 127 (S127), leading to its cytoplasmic retention, while phosphorylate at Serine 397 (S397) causes ubiquitination-dependent degradation [30]. Given that Tudor-SN diminishes the interaction between LATS1 and YAP, we speculated that Tudor-SN influences the phosphorylation and stability of YAP. The results demonstrated that the overexpression of Tudor-SN in myocardia of TG mice at P1 diminished the phosphorylation at S127 or S397 residue and increased the protein levels of YAP compared to P1 WT mice (Fig. 6A). Consistently, knockout of *Tudor-SN* in myocardia of *Myh6-Tudor-SN *^*−/−*^ mice enhanced the phosphorylation at S127 or S397 residue, and decreased the protein level of YAP compared to *Tudor-SN *^*fl/fl*^ mice (Fig. 6B). In addition, we overexpressed or knocked out Tudor-SN in H9c2 cells. Western blot analysis showed that both the protein and phosphorylation levels of YAP were influenced by the Tudor-SN protein levels in H9c2 cells (Fig. 6C-D). Then we investigated whether Tudor-SN influenced the stability of YAP using cycloheximide (CHX) treatment. As shown in Fig. 6E, YAP exhibited gradual degradation in H9c2 cells infected with pLVX-IRES-Flag vectors, whereas the degradation was noticeably retarded in Flag-Tudor-SN-H9c2 cells. Correspondingly, the degradation of YAP was promoted in *Tudor-SN*-KO-H9c2 cells compared to the WT H9c2 cells (Fig. 6F). Moreover, we detected the ubiquitylation of YAP by treating the cells with proteasome inhibitor MG132. The results showed a noticeable decrease in ubiquitylation of YAP in Flag-Tudor-SN-H9c2 cells compared to the vector-H9c2 cells (Fig. 6G), while there was a relative increase observed in *Tudor-SN*-KO-H9c2 cells compared to WT H9c2 cells (Fig. 6H).

We also investigated whether Tudor-SN influences the activity of the Hippo signaling pathway. The results showed no significant differences in protein and phosphorylation levels of the main components, MST1 (Macrophage stimulating 1) or LATS1, between WT and TG mice myocardia (Fig. S9A), as well as the *Tudor-SN *^*fl/fl*^ and *Myh6-Tudor-SN *^*−/−*^ mice myocardia (Fig. S9B). Meanwhile, there were no differences in H9c2 cells with gain- or loss-function of Tudor-SN compared to the control group (Fig. S9C-D). These findings indicate that Tudor-SN does not influence YAP phosphorylation by regulating the activity of the Hippo signaling pathway.

These results indicate that Tudor-SN interacts with YAP, preventing the phosphorylation induced by LATS1, subsequently prevents its ubiquitination and maintains its stability.

### Tudor-SN promotes the nucleus translocation and transcription factor activity of YAP

**Fig. 7:**
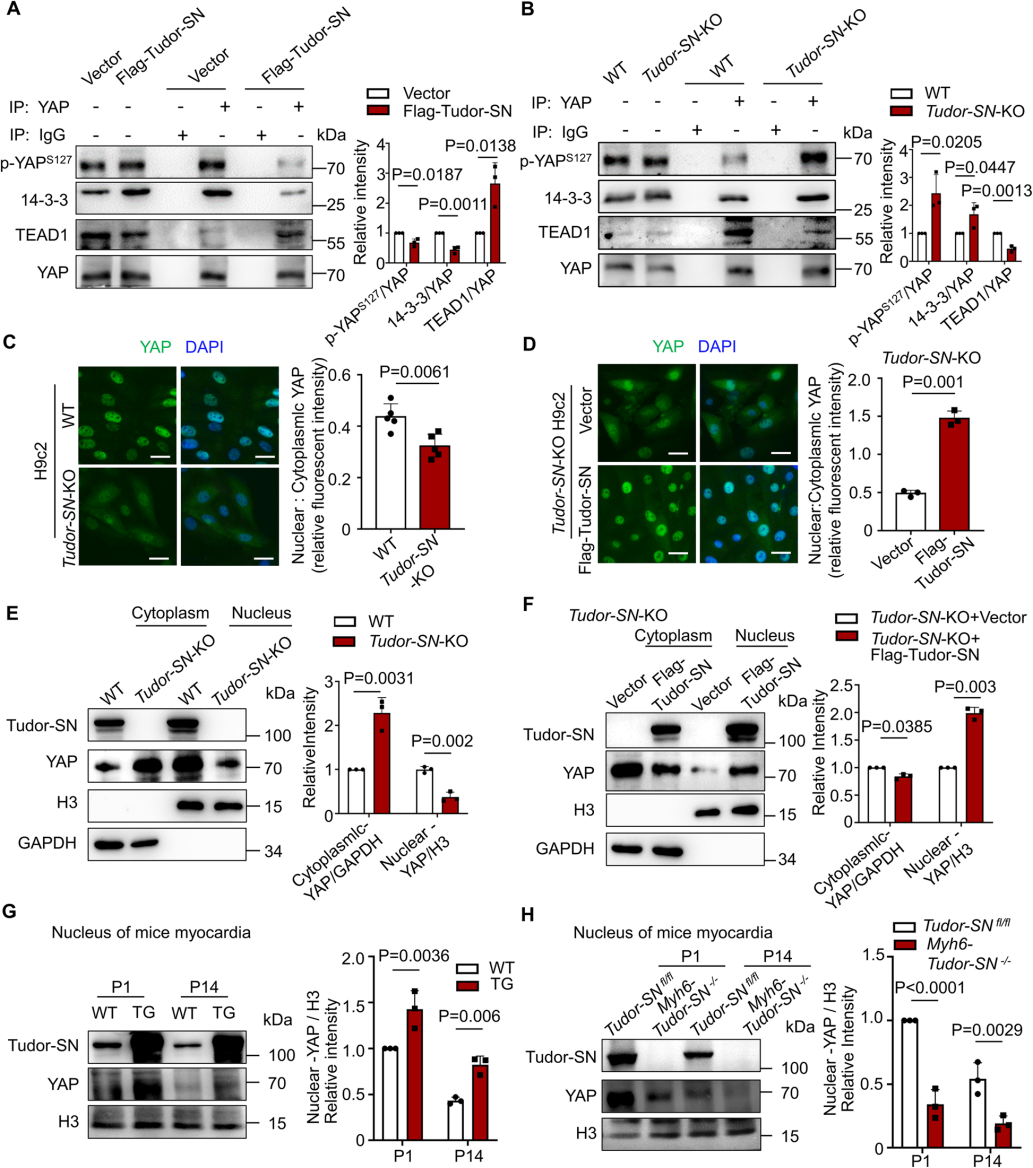
Tudor-SN promotes the nucleus translocation of YAP. **A**, **B** Total lysates of H9c2 cells transfected with Flag-vector or Flag-Tudor-SN, as well as WT and *Tudor-SN*-KO H9c2 cells were immunoprecipitated with anti-YAP antibodies. Western blot detected the level of YAP^S127^ and the interaction between YAP and 14-3-3 or TEAD1 (n = 3 biological replicates). **C** H9c2 WT and *Tudor-SN*-KO cells were immunostained with anti-YAP antibody (green) and counterstained with DAPI (blue) (Scale bar, 50 μm; n = 5 biological replicates). **D** H9c2 *Tudor-SN*-KO cells were rescued with Tudor-SN protein, Vector as a control. Then cells were immunostained with anti-YAP antibody and counterstained with DAPI (Scale bar, 50 μm; n = 3 biological replicates). **E**, **F** The cytoplasmic and nuclear proteins were isolated from WT and *Tudor-SN*-KO H9c2 cells, as well as *Tudor-SN*-KO H9c2 cells transfected with Flag-vector or Flag-Tudor-SN. Western blot detected YAP protein localization in cytoplasm and nucleus (n = 3 biological replicates). **G**, **H** The nuclear protein was isolated from WT and TG mice myocardia, as well as *Tudor-SN *^*fl/fl*^ mice and *Myh6-Tudor-SN *^*−/−*^ mice myocardia. Western blot detected YAP protein localization in nucleus (n = 3 biological replicates). The western blot results were analyzed by ImageJ (2 ×) software. All data were presented as the mean ± SEM, unpaired two-tailed Student’s t-test were used

Phosphorylation of YAP at S127 generates a binding anchor for 14-3-3 proteins, thereby facilitating the sequestration of YAP in the cytoplasm. Conversely, inhibition of YAP phosphorylation at S127 promotes its translocation into the nucleus and subsequent interaction with the transcription factor TEAD [31, 32]. Our results revealed that overexpression of Tudor-SN impaired the association between YAP and 14-3-3 while enhancing the YAP-TEAD interaction (Fig. 7A). Consistently, knockout of *Tudor-SN* enhanced the YAP-14-3-3 interaction but impaired the YAP-TEAD interaction (Fig. 7B). Immunostaining analysis further indicated that Tudor-SN protein affected the subcellular localization of YAP protein. As shown in Fig. 7C, *Tudor-SN*-KO H9c2 cells exhibited increased cytoplasmic distribution of YAP compared to WT H9c2 cells, whereas Tudor-SN restored cells displayed enhanced nuclear accumulation and reduced cytoplasmic presence of YAP when compared to *Tudor-SN*-KO cells (Fig. 7D). In order to further validate the correlation between the Tudor-SN protein and the nuclear translocation of YAP, we isolated the cytoplasmic and nuclear fractions to detect the subcellular distribution of YAP. As shown in Fig. 7E-F, more nuclear YAP and less cytoplasmic YAP were detected in WT H9c2 cells compared to *Tudor-SN*-KO H9c2 cells, while less nuclear YAP and more cytoplasmic YAP in *Tudor-SN*-KO H9c2 cells compared with Tudor-SN restored cells. To corroborate these findings, we also examined myocardial samples from WT and TG mice at P1 and P14, as well as *Tudor-SN *^*fl/fl*^ and *Myh6-Tudor-SN *^*−/−*^ mice. Consistently, the results from mouse myocardia supported that Tudor-SN promotes the nucleus translocation of YAP (Fig. 7G-H). Immunoblotting analysis of H3 or GAPDH demonstrated equal amount of nuclear or cytoplasmic proteins were loaded across all samples.

To investigate the impact of Tudor-SN on YAP function, we performed qRT-PCR to detect the mRNA levels of several YAP target genes (*Aurk B, Cdc45, Ccna1, Ccne1, Cdk6* and *E2f1*) associated with cell proliferation [20, 33, 34] in myocardia tissues from WT and TG-mice, as well as *Tudor-SN *^*fl/fl*^ mice and *Myh6-Tudor-SN *^*−/−*^ mice at P1. The mRNA levels of *Aurk B, Cdc45, Ccna1, Ccne1, Cdk6* and *E2f1* were higher in TG mice myocardia compared to WT mice (Fig. S10A), while lower in *Myh6-Tudor-SN *^*−/−*^ mice myocardia compared to *Tudor-SN *^*fl/fl*^ mice (Fig.S10B). Similar results were obtained in H9c2 WT and *Tudor-SN*-KO cells (Fig. S10C).

These results indicate that Tudor-SN can facilitate the nuclear translocation of YAP leading to enhanced transcriptional activity of its downstream proliferation-related genes.

## Discussion

Investigating the mechanisms underlying neonatal cardiomyocyte proliferation and regeneration is crucial for overcoming the limited regenerative capacity of adult mammalian cardiomyocytes. In the present study, we have identified that Tudor-SN as a novel regulator of neonatal cardiomyocyte proliferation. Our findings demonstrate that Tudor-SN influences the phosphorylation of YAP, thereby maintaining stability and facilitating nuclear translocation of YAP to promote and prolong neonatal cardiomyocyte proliferation under physiological conditions, and enhance neonatal heart regeneration following AR injury.

We previously identified Tudor-SN as a cell cycle regulator that promoting tumor cell proliferation [16]. Here, we first describe the role of Tudor-SN in promoting the cardiomyocyte proliferation. Tudor-SN enhances the proliferation ability of cardiomyocyte not only during the immediate neonatal period but also beyond the postnatal two weeks when most cardiomyocytes normally exit the cell cycle and enter a more differentiated phase coincident with hypertrophic growth and bi-nucleation. Moreover, our findings demonstrate that Tudor-SN does not affect the size and apoptosis of cardiomyocytes, providing further evidence that Tudor-SN influences the heart size and function mainly through regulating the cardiomyocyte proliferation.

Non-cardiomyocytes, such as cardiac fibroblasts, endothelial cells, and immune cells, also play important roles in regulating cardiac regeneration and cardiomyocyte proliferation. As the *Tudor-SN* transgenic mouse is not heart specific, we detected the influence of Tudor-SN on the non-cardiomyocytes. However, no significant responses to Tudor-SN were observed in fibroblasts and endothelial cells proliferation. The variable effects on proliferation among different cell types may be related to the varying expression levels of Tudor-SN and the specific cellular environments in different cell types. Studies have demonstrated that non-cardiomyocytes secrete factors that regulate cardiomyocyte proliferation. For example, the fibroblasts release Versican, an extracellular matrix component, after neonatal myocardial injury and promote cardiomyocyte proliferation and cardiac repair [35]. Macrophages promote cardiomyocyte proliferation by secreting oncostatin M during neonatal heart regeneration [25]. Although we excluded the influence of Tudor-SN on fibroblasts and endothelial cells proliferation, regretfully, we could not completely rule out the influence from secreted factors of these cells. Future studies are required to explore the influence of Tudor-SN on non-cardiomyocyte behavior and the interplay between different cells in the heart.

The Hippo-YAP pathway participates in heart development and repairment by regulating the proliferation and regeneration of cardiomyocytes [36]. YAP forms the complex with the transcription factor TEAD, thereby controlling the transcriptional activity of target genes, particularly those involved in cell cycle regulation and cell growth [37]. When the Hippo pathway is activated, YAP protein is phosphorylated by LATS1/2 at various serine residues, leading to its cytoplasmic retention, ubiquitination and subsequent proteolytic degradation [38]. Based on the theory, an effective strategy to enhance cardiomyocyte proliferation is loss-function of Hippo pathway or consistent activation of YAP. For instance, a series of studies have verified that cardiomyocyte-specific deletion or inhibition the activity of the key components in the Hippo pathway (such as MST1/2, LATS1/2) [39, 40], as well as over-expression of a constitutively active mutant YAP [41, 42], are sufficient to promote cardiomyocyte proliferation and inducing re-entry into the cell cycle in both neonatal and adult mouse hearts. In this present study, we reveal that Tudor-SN regulates phosphorylation of YAP and facilitates neonatal cardiomyocyte proliferation and regeneration. Our results show that Tudor-SN exerts no influence on the activity of Hippo signaling pathway, but rather impedes the association between LATS1 and the WW domain of YAP. As a result, phosphorylation at S127 and S397 mediated by LATS1 are inhibited. Correspondingly, there is a reduction in the interaction between YAP and 14-3-3 protein, leading to diminish cytoplasmic retention as well as ubiquitination-dependent degradation, whereas the nuclear translocation and the interaction with transcription factor TEAD are enhanced. Our study identifies Tudor-SN as a novel regulator of YAP and unveils a new model for regulating YAP activity.

The expression of Tudor-SN is significantly higher in rapidly proliferating neonatal cardiomyocytes compared to terminally differentiated adult cardiomyocytes [17]. Coincidentally, previous studies have shown that the level of YAP is robustly detected in neonatal mouse cardiomyocytes and gradually declined with advancing age, becoming nearly undetectable in adult cardiomyocytes [20]. In this study, we observed that Tudor-SN upregulates the protein level of YAP in P1 neonatal mouse myocardia, but not in adult mouse myocardia (Fig. S11A). Furthermore, we found a significant decrease in the mRNA level of YAP during heart development (Fig. S11B). It interprets the high expression of Tudor-SN protein in TG-mice does not influence the protein level of YAP in adult mouse myocardia, consequently insufficient to retain the cell proliferation in adult cardiomyocyte. This study also reminds us that simultaneously upregulating Tudor-SN and YAP protein level is a potential strategy to promote the adult cardiomyocytes re-enter cell cycle and repair the cardiac injury.

mTOR and Hippo-YAP are two major signaling pathways that collaborate to regulate the organ development through their respective roles in control of cell growth and cell number [43]. Studies over the past years have revealed the functional links between the mTOR and Hippo-YAP pathways. For instance, mTOR can increase the accumulation of YAP through inhibiting the autophagosome and lysosome system, thereby improving its target genes expression [44], while LATS phosphorylates Raptor to attenuate mTORC1 activation by impairing the interaction of Raptor with Rheb [45]. Our previous study showed that the expression of Tudor-SN was controlled by the mTORC1 signaling pathway and is involved in the mTORC1-mediated regulation of cardiomyocytic proliferation [17]. In the present study, we demonstrated that Tudor-SN regulates the phosphorylation of YAP, thereby promotes neonatal cardiomyocytes proliferation and regeneration. These results imply that Tudor-SN may serve as a novel functional link between the mTORC1 and YAP pathway, consequently involving in the regulation of cardiomyocytes proliferation and regeneration.

## Conclusion

In summary, Tudor-SN functions as a novel regulator that modulates the phosphorylation of YAP, thereby promoting transcription of proliferation- related genes consequently promotes and prolongs neonatal cardiomyocyte proliferation under physiological conditions and facilitates neonatal heart regeneration following AR injury.

### Supplementary Information

Additional file 1: Fig. S1 Construction of *Tudor-SN* transgenic (TG) mice. Fig. S2 Overexpression of Tudor-SN promotes neonatal cardiomyocyte proliferation in vitro and in vivo. Fig. S3 Overexpression of Tudor-SN does not influence the cardiomyocytes size, cardiac fibrosis, and the proliferation of fibroblasts and endothelial cells. Fig. S4 The flow cytometry gating strategy and the number of cardiomyocytes, fibroblasts, and endothelial cells. Fig. S5 Construction of *Myh6-Tudor-SN*^*−/−*^ mice. Fig. S6 Knockout of *Tudor-SN* inhibits neonatal cardiomyocytes proliferation in vitro and in vivo. Fig. S7 Knockout of *Tudor-SN* does not influence the cardiac fibrosis, cardiomyocytes size and apoptosis. Fig. S8 Coomassie blue staining for GST-fusion proteins. Fig. S9 The activity of Hippo pathway is not affected by Tudor-SN. Fig. S10 Tudor-SN increases the mRNA level of YAP downstream proliferation-related genes. Fig. S11 Tudor-SN upregulates the protein level of YAP in P1 but not P28 mouse myocardia. Table S1 Primers sequences for Genotyping. Table S2 Primer sequences for sgRNAs. Table S3 Primer sequences for Tudor-SN and YAP plasmids. Table S4 Antibody information. Table S5 qRT-PCR-primer sequences. Table S6 Primer sequences of GST plasmids

Additional file 2. Uncut gel blot.

## Data Availability

No datasets were generated or analysed during the current study.
