# Supplementary material for: Tudor-SN promotes cardiomyocyte proliferation and neonatal heart regeneration through regulating the phosphorylation of YAP
Source: Cell Commun Signal. 2024 Jun 28;22:345. doi: 10.1186/s12964-024-01715-6 (PMC11212424; doi:10.1186/s12964-024-01715-6)
Supplement: Supplementary file 1 — Additional file 1: Fig. S1 Construction of Tudor-SN transgenic (TG) mice. Fig. S2 Overexpression of Tudor-SN promotes neonatal cardiomyocyte proliferation in vitro and in vivo. Fig. S3 Overexpression of Tudor-SN does not influence the cardiomyocytes size, cardiac fibrosis, and the proliferation of fibroblasts and endothelial cells. Fig. S4 The flow cytometry gating strategy and the number of cardiomyocytes, fibroblasts, and endothelial cells. Fig. S5 Construction of Myh6-Tudor-SN−/− mice. Fig. S6 Knockout of Tudor-SN inhibits neonatal cardiomyocytes proliferation in vitro and in vivo. Fig. S7 Knockout of Tudor-SN does not influence the cardiac fibrosis, cardiomyocytes size and apoptosis. Fig. S8 Coomassie blue staining for GST-fusion proteins. Fig. S9 The activity of Hippo pathway is not affected by Tudor-SN. Fig. S10 Tudor-SN increases the mRNA level of YAP downstream proliferation-related genes. Fig. S11 Tudor-SN upregulates the protein level of YAP in P1 but not P28 mouse myocardia. Table S1 Primers sequences for Genotyping. Table S2 Primer sequences for sgRNAs. Table S3 Primer sequences for Tudor-SN and YAP plasmids. Table S4 Antibody information. Table S5 qRT-PCR-primer sequences. Table S6 Primer sequences of GST plasmids [file 12964_2024_1715_MOESM1_ESM.pdf]

Additional file 1.

Tudor-SN promotes cardiomyocyte proliferation and neonatal heart regeneration through regulating the phosphorylation of YAP

Chao Su[2 †], Jinzheng Ma[1 †], Xuyang Yao[1, 3 †], Wei Hao[1], Shihu Gan[1], Yixiang Gao[1], Jinlong He[1], Yuanyuan Ren[1], Xingjie Gao[1], Yi Zhu[1], Jie Yang [1 *]and Minxin Wei[2 *]

[1] Tianjin Key Laboratory of Cellular and Molecular Immunology, Key Laboratory of Immune Microenvironment and Disease (Ministry of Education), State Key Laboratory of Experimental Hematology, Tianjin Key Laboratory of Metabolic Diseases, The Province and Ministry Co-sponsored Collaborative Innovation Center for Medical Epigenetics, Tianjin Medical University, Tianjin, China.

[2] Division of Cardiovascular Surgery, Cardiac and Vascular Center, the University of Hong Kong-Shenzhen Hospital, Shenzhen, China.

[3] Department of Ophthalmology, Tianjin Medical University General Hospital, Tianjin, China.

* Correspondence:

E-mail: yangj@tmu.edu.cn (J.Y.), weimx@hku-szh.org (MX.W.)

[†] Chao Su, Jinzheng Ma and Xuyang Yao contributed equally to this work.

Supplementary Figures

Figure S1

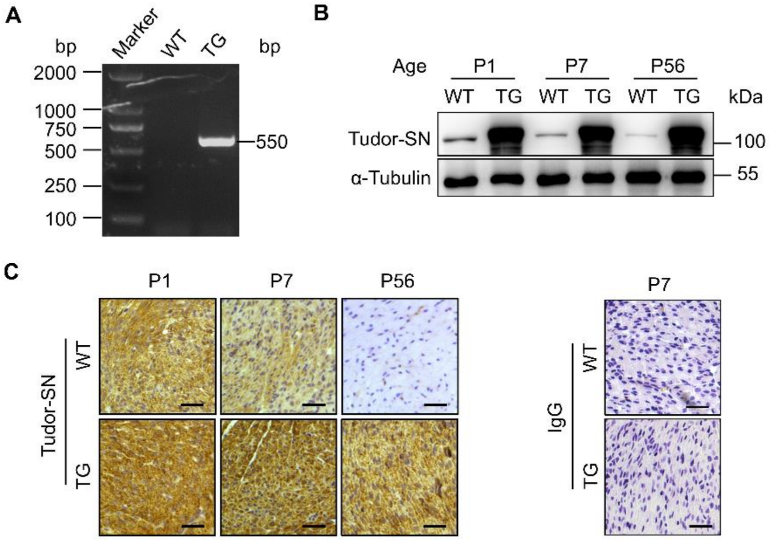

Figure S1. Construction of *Tudor-SN* transgenic (TG) mice. (A) Genotyping of Wild Type (WT) and Tudor-SN transgenic (TG) mice. **(B)** Tudor-SN expression levels in myocardia of WT and TG mice at P1, P7 and P56 were detected by western blot. **(C)** Representative immunohistochemical staining with anti-Tudor-SN antibody in myocardia of WT and TG mice at P1, P7 and P56 (Scale bar, 50 μm).

Figure S2

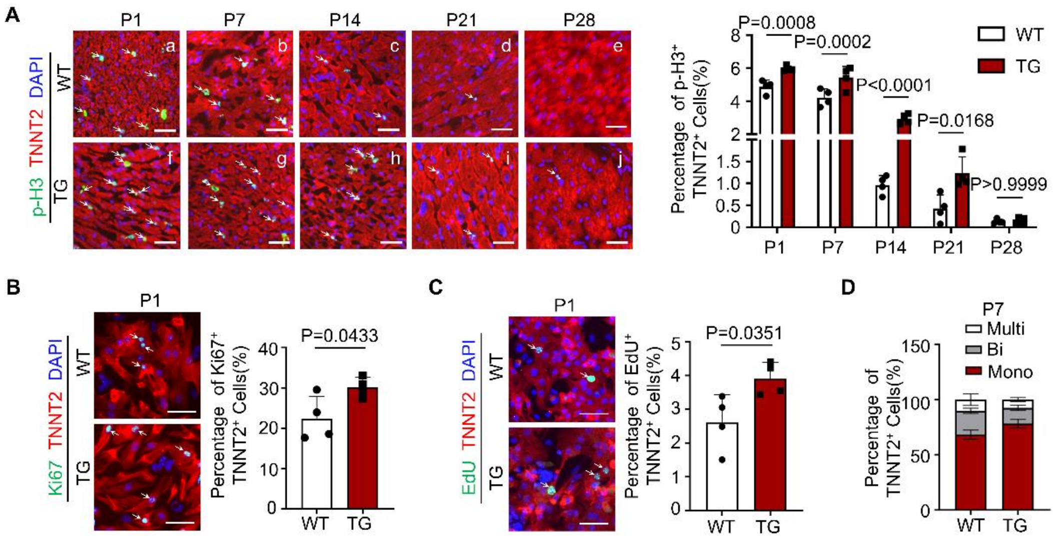

Figure S2. Overexpression of Tudor-SN promotes neonatal cardiomyocyte proliferation *in vitro* and *in vivo*. (A) Myocardia tissues from postnatal day 1 (P1), P7, P14, P21 and P28 WT and TG mice were sliced and subjected to immunofluorescence staining with anti-p-H3 (green), anti-TNNT2 (red) antibodies and DAPI (blue). Representative immunostaining and percentage of p-$H3^+$ $TNNT2^+$ cells were shown (Scale bar, 50 μm; n = 4 biological replicates). **(B)** Primary cardiomyocytes were isolated from P1 WT and TG mice, then immunostained with anti-Ki67 antibody (green), anti-TNNT2 antibody (red) and counterstained with DAPI (blue) (Scale bar, 50 μm; n = 4 biological replicates). **(C)** Primary cardiomyocytes from P1 WT and TG mice were incorporated with EdU (green) and stained with DAPI (blue). Representative immunostaining and percentage of EdU^+ $TNNT2^+$ cells were shown (Scale bar, 50 μm; n = 4 biological replicates). **(D)** The percentage of multi-, bi- and mono-nucleus cardiomyocytes of WT and TG mice at P7 were shown (n = 4 biological replicates). All data were presented as the mean ± SEM, results in **A** were analyzed by repeated measures ANOVA followed by Bonferroni post-hoc correction, unpaired two-tailed Student's t-test were used in **B-D**.

Figure S3

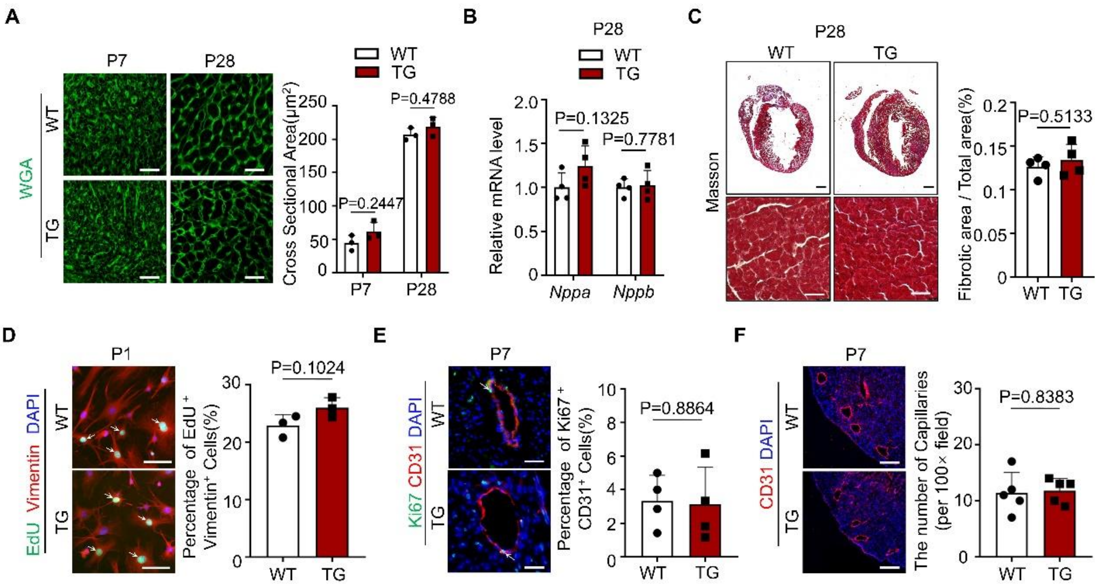

Figure S3. Overexpression of Tudor-SN does not influence the cardiomyocytes size, cardiac fibrosis, and the proliferation of fibroblasts and endothelial cells. (A) Wheat germ agglutinin (WGA) staining of the myocardia of WT and TG mice at P7 and P28 (Scale bar, 50μm; n = 3 biological replicates). **(B)** qRT-PCR detected the mRNA level of *Nppa* and *Nppb* in myocardia of WT and TG mice at P28 (n = 4 biological replicates). **(C)** Masson's trichrome staining of the P28 WT and TG mice myocardia (Scale bar, 1mm; 100μm; n = 4 biological replicates). **(D)** Primary cardiac fibroblasts were incorporated with EdU (green), and stained with anti-Vimentin antibody (red) and DAPI (blue). Percentage of EdU^{+} $Vimentin^{+}$ cells were shown (Scale bar, 50 μm; n = 3 biological replicates). **(E)** The myocardia of WT and TG mice at P7 were stained with anti-Ki67 (green), anti-CD31 antibody (red) and DAPI (blue). Percentage of $Ki67^{+}$ $CD31^{+}$ cells were shown (Scale bar, 50 μm; n = 4 biological replicates). **(F)** The P7 WT and TG mice myocardia were immunofluorescence stained with anti-CD31 antibody (red) and DAPI (blue). Representative immunostaining and number of capillaries per field (100×) were shown (Scale bar, 200 μm; n = 5 biological replicates). All data were presented as the mean ± SEM, unpaired two-tailed Student's t-test were used in **A-F**.

Figure S4

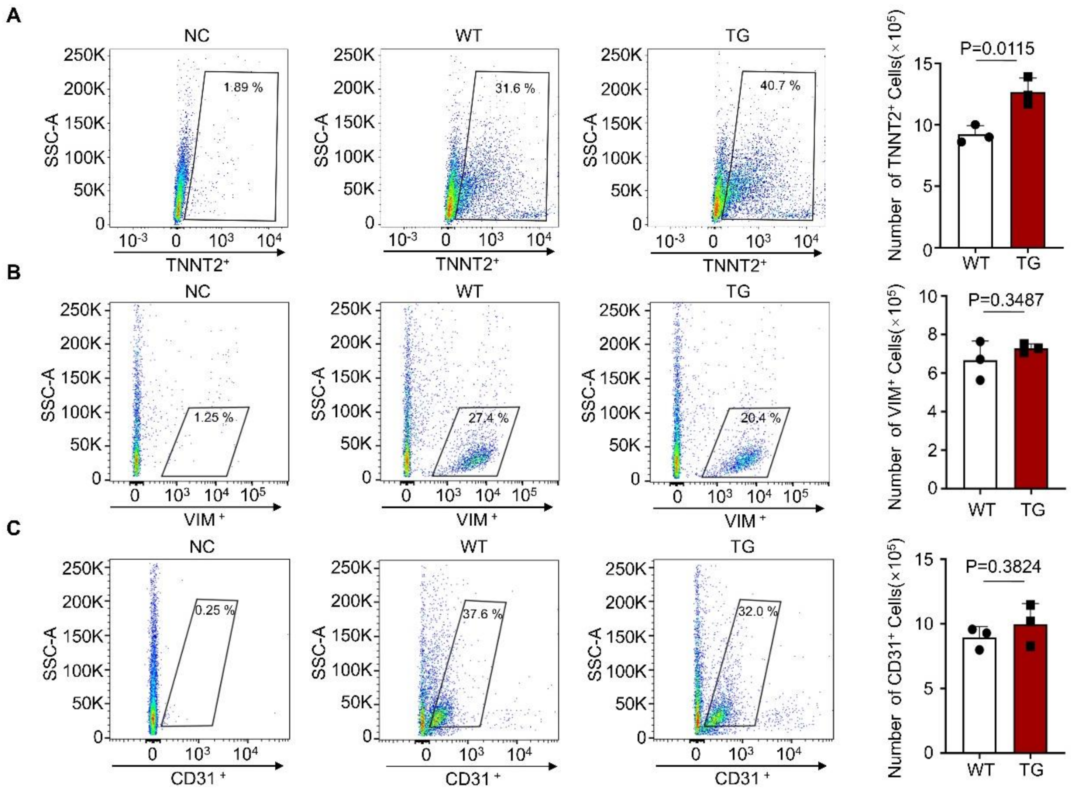

Figure S4. The flow cytometry gating strategy and the number of cardiomyocytes, fibroblasts, and endothelial cells. Total cell suspensions from the heart of WT and TG mice at P7 were prepared and counted by cell counter. **(A-C)** Cardiomyocytes, fibroblast and endothelial cells were labeled with anti-TNNT2, anti-Vimentin (VIM) and anti-CD31 antibodies respectively, and sorted by flow cytometry. The number of each specific cell type equals the total number of cells multiplied by the percentage of each specific cell. (n = 3 biological replicates). All data were presented as the mean ± SEM, unpaired two-tailed Student's t-test were used.

Figure S5

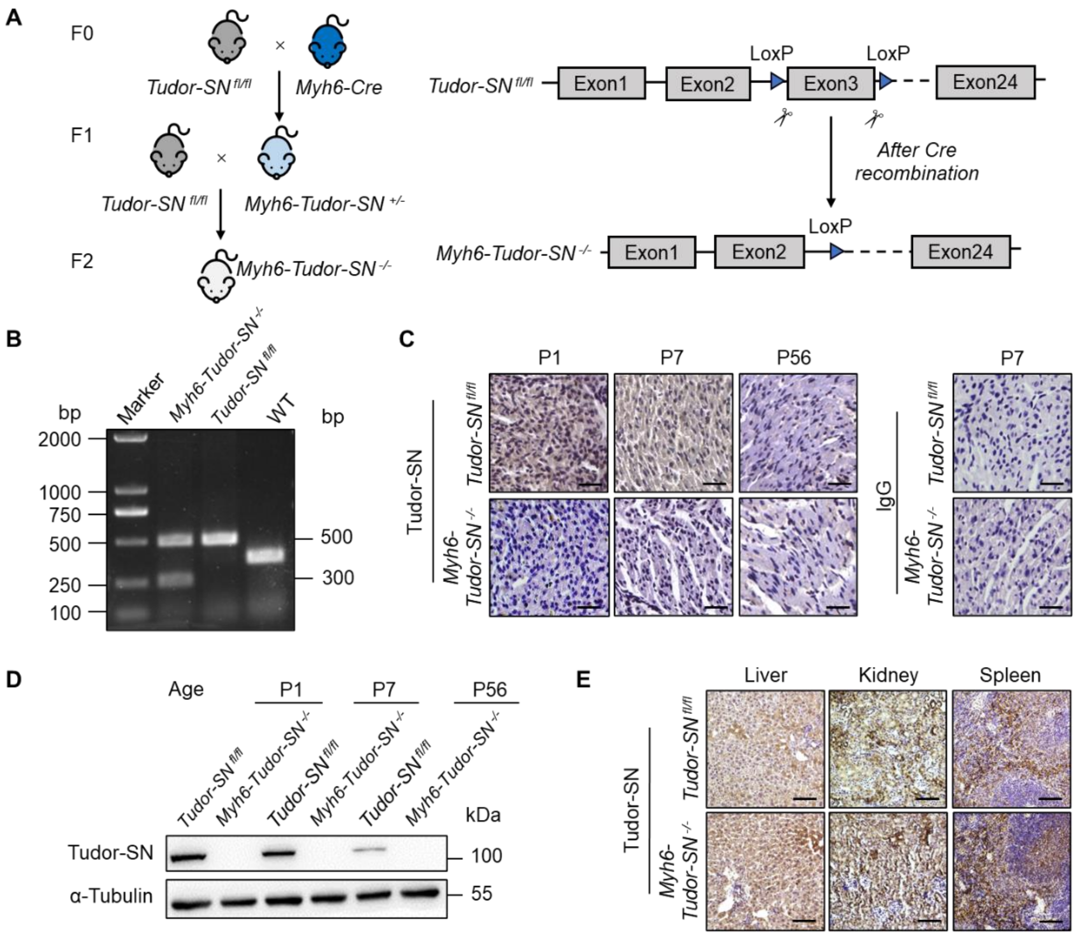

Figure S5. Construction of *Myh6-Tudor-SN* $^{-/-}$ mice. (A) Schematic representation of *Myh6-Tudor-SN* $^{-/-}$ mice generation. **(B)** Genotyping of *Myh6-Tudor-SN* $^{-/-}$, *Tudor-SN* $^{fl/fl}$ and WT mice. **(C)** Representative immunohistochemical staining with anti-Tudor-SN antibody in myocardia of *Tudor-SN* $^{fl/fl}$ and *Myh6-Tudor-SN* $^{-/-}$ mice at P1, P7 and P56 (Scale bar, 50 μm). **(D)** Tudor-SN expression level in myocardia of *Tudor-SN* $^{fl/fl}$ and *Myh6-Tudor-SN* $^{-/-}$ mice at P1, P7 and P56 were detected by western blot. **(E)** Immunohistochemical staining of Tudor-SN in liver, kidney, and spleen tissue of *Tudor-SN* $^{fl/fl}$ and *Myh6-Tudor-SN* $^{-/-}$ (Scale bar, 50 μm).

Figure S6

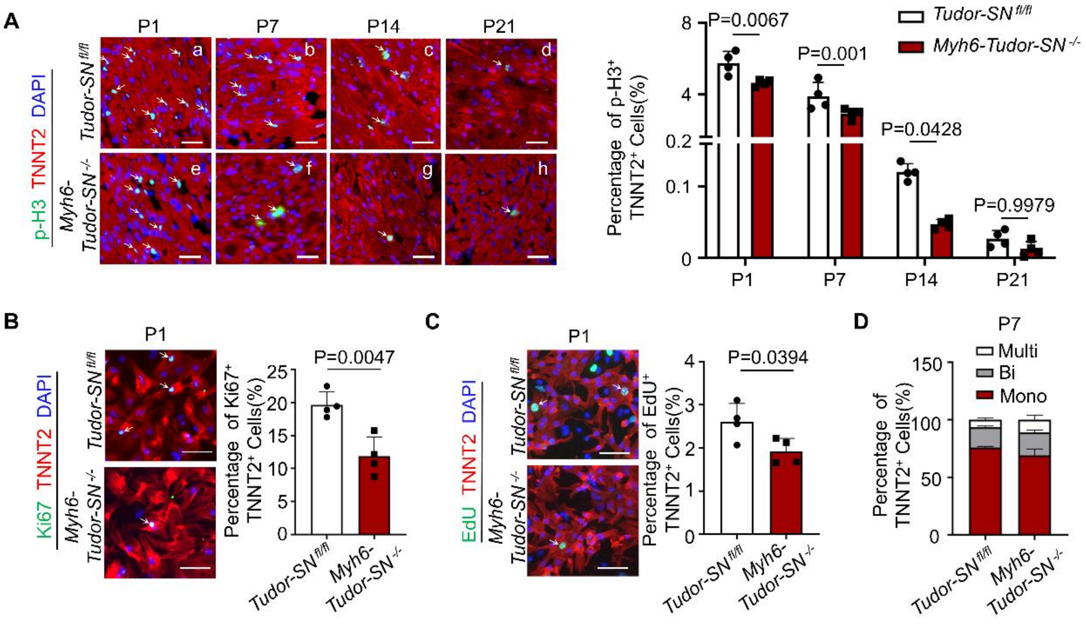

Figure S6. Knockout of *Tudor-SN* inhibits neonatal cardiomyocytes proliferation *in vitro* and *in vivo*. (A) The *Tudor-SN* $^{fl/fl}$ and *Myh6-Tudor-SN* $^{-/-}$ mice myocardia were immunofluorescence stained with anti- p-H3 (green), anti-TNNT2 antibody (red) and DAPI (blue). The percentage of p-H3^{+} TNNT2^{+} cells were shown (Scale bar, 50 μm; n = 4 biological replicates). **(B)** Primary cardiomyocytes from P1 WT and TG mice were immunostained with anti-Ki67 antibody (green), anti-TNNT2 antibody (red) and counterstained with DAPI (blue). The percentage of Ki67^{+} TNNT2^{+} cells were shown (Scale bar, 50 μm; n = 4 biological replicates). **(C)** Primary cardiomyocytes from P1 WT and TG mice were incorporated with EdU (green) and stained with DAPI (blue). Representative immunostaining and percentage of EdU^{+} TNNT2^{+} cells were shown (Scale bar, 50 μm; n = 4 biological replicates). **(D)** The percentage of multi-, bi- and mono-nucleus cardiomyocytes of WT and TG mice at P7 were shown (n = 4 biological replicates). All data were presented as the mean ± SEM, results in **A** were analyzed by repeated measures ANOVA followed by Bonferroni post-hoc correction, unpaired two-tailed Student's t-test were used in **B-D**.

Figure S7

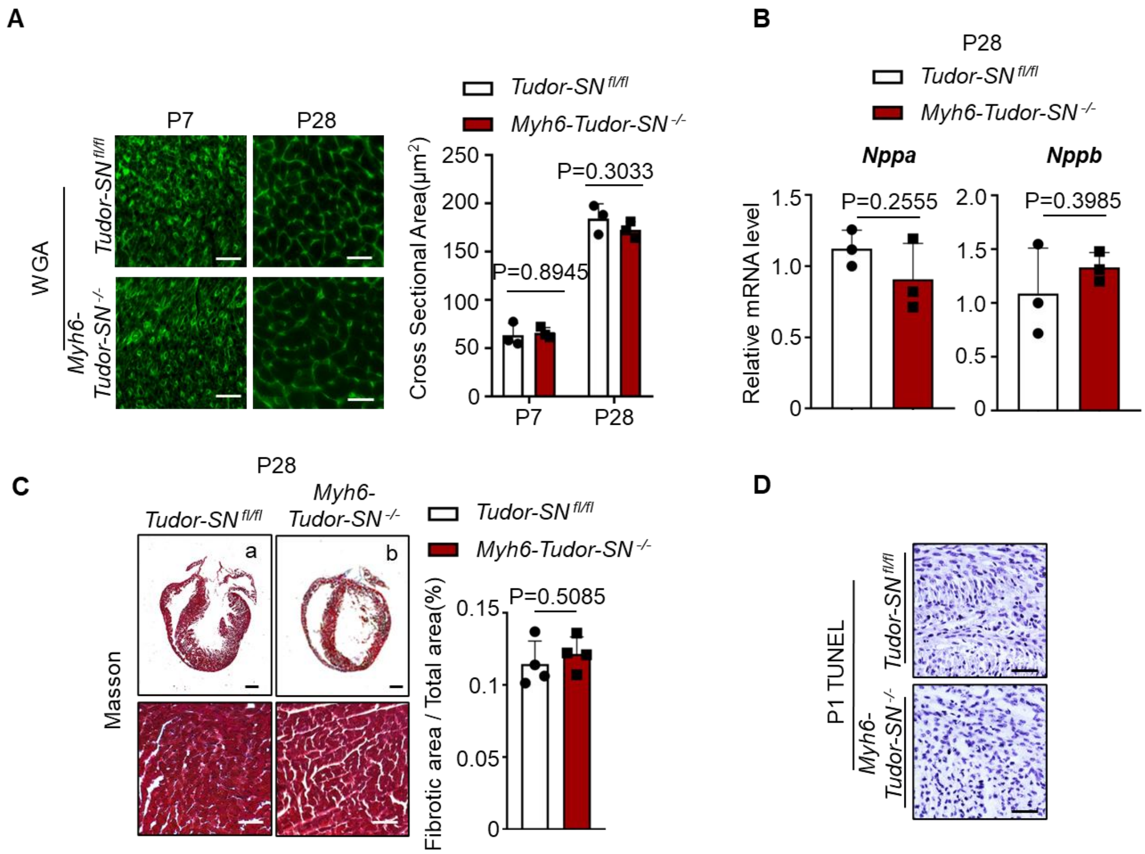

Figure S7. Knockout of *Tudor-SN* does not influence the cardiac fibrosis, cardiomyocytes size and apoptosis. (A) Wheat germ agglutinin (WGA) staining of the myocardia of *Tudor-SN* $^{fl/fl}$ and *Myh6-Tudor-SN* $^{-/-}$ mice at P7 and P28 (Scale bar, 50μm; n = 3 biological replicates). **(B)** qRT-PCR detected the mRNA level of *Nppa* and *Nppb* in *Tudor-SN* $^{fl/fl}$ and *Myh6-Tudor-SN* $^{-/-}$ mice myocardia at P7 (n = 3 biological replicates). **(C)** Masson's trichrome staining of the *Tudor-SN* $^{fl/fl}$ and *Myh6-Tudor-SN* $^{-/-}$ mice myocardia at P28 (Scale bar, 1mm; Scale bar, 100μm). **(D)** Apoptosis of *Tudor-SN* $^{fl/fl}$ and *Myh6-Tudor-SN* $^{-/-}$ mice cardiomyocytes was detected by TUNEL assay (Scale bar, 50 μm). Representative immunostaining were shown. All data were presented as the mean ± SEM, unpaired two-tailed Student's t-test were used in **A-C**.

Figure S8

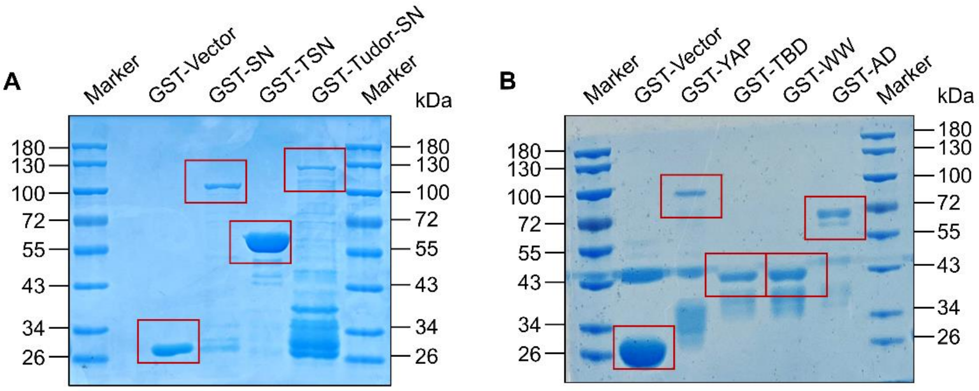

Figure S8. Coomassie blue staining for GST-fusion proteins. (**A**) The result of Coomassie blue staining for GST-fusion proteins matches **Figure 5D**. (**B**) The result of Coomassie blue staining for GST fusion proteins matches **Figure 5E**.

Figure S9.

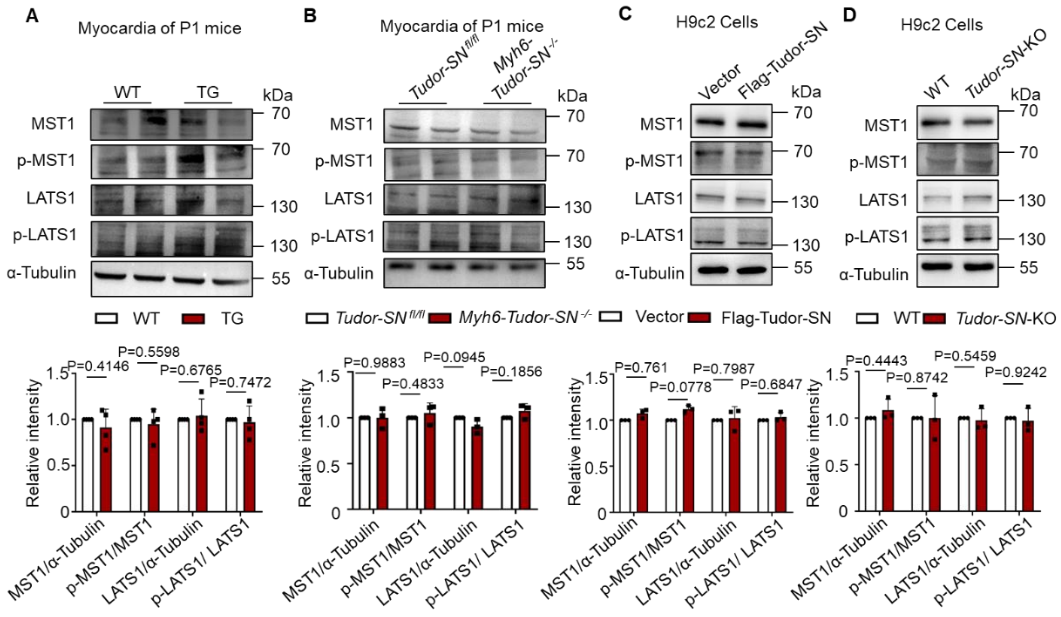

Figure S9. The activity of Hippo pathway is not affected by Tudor-SN. **(A-B)** The expression levels of Tudor-SN, MST1, p-MST1, LATS1, p-LATS1 and α-Tubulin in myocardia of WT and TG mice, as well as *Tudor-SN*$^{fl/fl}$ and *Myh6-Tudor-SN* $^{-/-}$ mice at P1 were detected by western blot (n = 4 biological replicates). **(C-D)** The expression levels of Tudor-SN, MST1, p-MST1, LATS1 and p-LATS1 in H9c2 cells transfected with Flag-vector or Flag-Tudor-SN, as well as WT or *Tudor-SN*-KO H9c2 cells were detected by western blot (n = 3 biological replicates). All data were presented as the mean ± SEM, unpaired two-tailed Student's t-test were used.

Figure S10

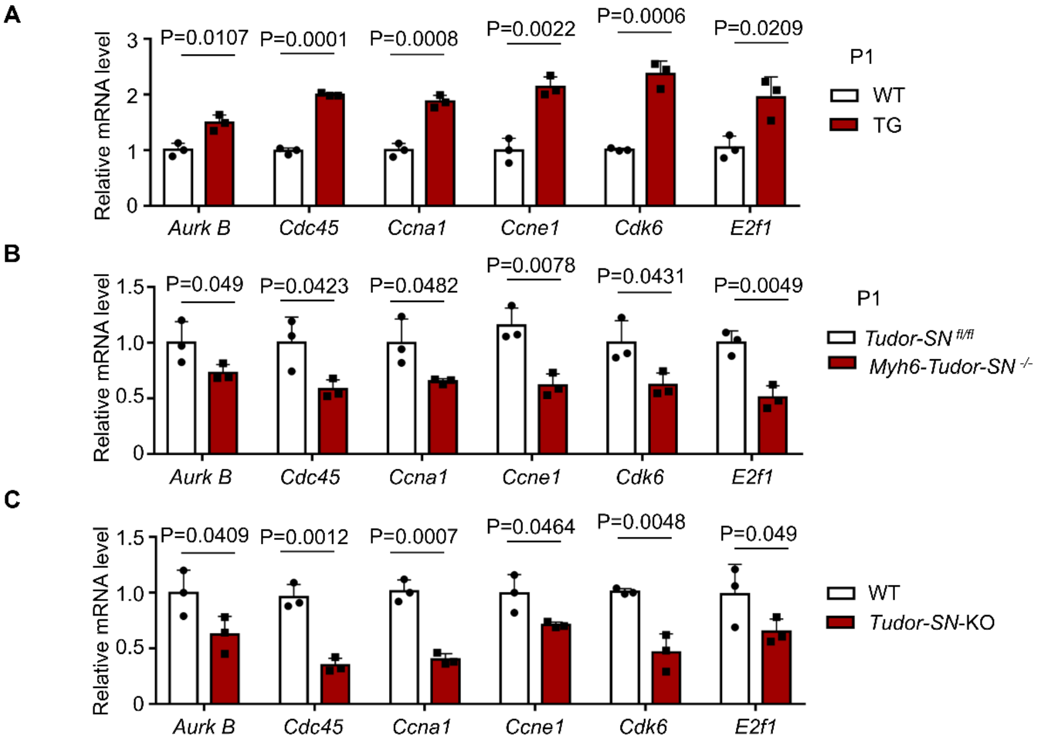

Figure S10. Tudor-SN increases the mRNA level of YAP downstream proliferation-related genes. (A) The qRT-PCR analyze of the gene expression of YAP downstream targets in P1 WT and TG mice myocardia (n = 3 biological replicates). **(B)** The relative mRNA levels of YAP downstream genes in P1 *Tudor-SN*$^{fl/fl}$ and *Myh6-Tudor-SN* $^{-/-}$ mice myocardia (n = 3 biological replicates). **(C)** The relative mRNA levels of YAP downstream genes in H9c2 WT and *Tudor-SN*-KO cells (n = 3 biological replicates). All data were presented as the mean ± SEM, , unpaired two-tailed Student's t-test were used.

Figure S11.

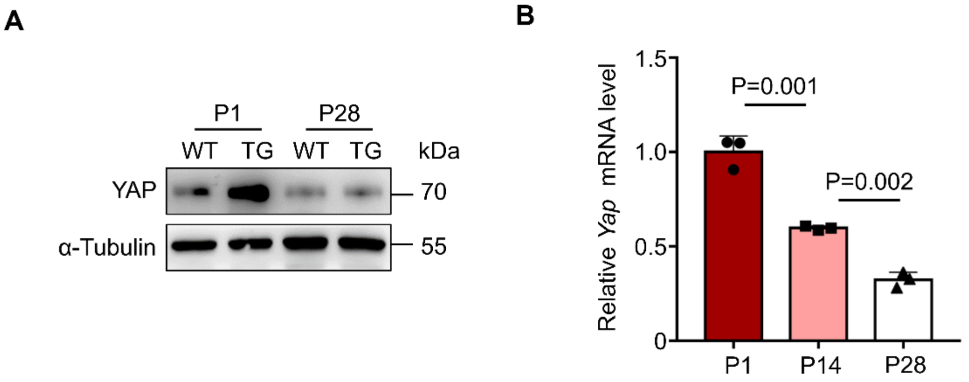

Figure S11. Tudor-SN upregulates the protein level of YAP in P1 but not P28 mouse myocardia. (A) YAP expression levels in myocardia of WT and TG mice at P1 and P28 were detected by western blot. **(B)** The qRT-PCR analyzed the relative mRNA levels of YAP at P1, P14, P28 mice myocardia (n = 3 biological replicates). Data were presented as the mean ± SEM, result in **B** was analyzed by one-way ANOVA followed by Bonferroni post-hoc correction.

Supplementary Tables

Table S1

Table S1 Primers sequences for Genotyping

Gene	Primer
Flox-Forward primer	CAGCACTAAAAGCTTGTCCC
Flox-Reverse primer	GCTAAAGAGTCCCTAGAAAG
Myh6-Cre-Forward primer	CTCCTTCCAGTCCACAAACGACC
Myh6-Cre-Reverse primer	GGCGATCCCTGAACATGTCC
TG-Forward primer	GCAACGTGCTGGTTATTGTG
TG-Reverse primer	CTGGGTTGTTGGCTCTCAT

Table S2

Table S2 Primer sequences for sgRNAs

Gene	Primer
sgRNAs-α-Forward primer	CACCGTCTGGAGGCGGACCGCCCCG
sgRNAs-α-Reverse primer	CAGACCTCCGCCTGGCGGGGCCAAA
sgRNAs-β-Forward primer	CACCGAGCTGGAAATCTCGCCCGT
sgRNAs-β-Reverse primer	CTCGACCTTTAGAGCGGGCACAAA

Table S3

Table S3 Primer sequences for Tudor-SN and Yap plasmids

Gene	Primer
Flag-*Tudor-SN*-Forward primer	CGCGGATCCAATGAATAGCAGCAGTAGGAACATTAGGTAGCGACTGTAGCCAAACTCATCA
Flag-*Tudor-SN*-Reverse primer	CCGCTCGAGATGGCCTCCGCGCAGAGCAGCGGC
Flag-*Yap*-Forward primer	CCGGAATTCATGGATTACAAGGATGACGACGATAAGATGGAGCCCGCGCAACAGCC
Flag-*Yap*-Reverse primer	CGCGGATCCCTATTGGTTGTCATTGTCCT

Table S4

Table S4 Antibody information

Antibody	Corporation	Catalog	Working concentration
anti-cTNT	Abcam	ab8295	IF: 1:100; Flow Cyt: 1:100
anti-Flag	Abcam	ab205606	WB: 1:1000
anti-GAPDH	Proteintech	60004-1-Ig	WB: 1:1000
anti-H3	CST	#4499	WB: 1:1000
anti-Ki67	Thermo Fisher Scientific	PA5-19462	IF: 1:100
anti-Aurora B	Sigma-Aldrich	A5102	IF: 1:100
anti-Vimentin	Abcam	ab92547	IF: 1:100; Flow Cyt: 1:100
anti-CD31	Abcam	ab9498	IF: 1:100; Flow Cyt: 1:100
anti-LATS1	CST	#3477	WB: 1:1000
anti-MST1	Abcam	ab51134	WB: 1:1000
anti-p-H3	Thermo Fisher Scientific	PA5-17869	IF: 1:100
anti-p-LATS1 (Thr1079)	CST	#8654	WB: 1:1000
anti-p-MST1/2 (pThr183)	Sigma-Aldrich	SAB4504042	WB: 1:1000
anti-S127-pYAP	CST	#4911	WB: 1:1000
anti-S397-pYAP	Proteintech	29018-1-AP	WB: 1:1000
anti-Tudor-SN	Abcam	ab65078	WB: 1:1000; IHC: 1:200; IP: 5μg/ml
anti-UBIQUITIN	Proteintech	10201-2-AP	WB: 1:2000
anti-YAP	CST	#14074	WB: 1:1000; IF: 1:100; IP:5μg/ml
anti-α-Tubulin	Sigma-Aldrich	T5168	WB: 1:1000
anti-14-3-3 Epsilon	Novus Biologicals	NBP1-32695	WB: 1:1000
anti-TEAD1	Abcam	ab133533	WB: 1:1000

Table S5

Table S5 qRT-PCR-primer sequences

Tudor-SN (Mouse)	Forward primer	TGTGCCACTGTCACCATTGGAG
	Reverse primer	CAGCTCATCGTAGTGTGAAGACC
Gapdh (Mouse)	Forward primer	CATCACTGCCACCCAGAAGACTG
	Reverse primer	ATGCCAGTGAGCTTCCCGTTCAG
Aurkb (Mouse)	Forward primer	CTTCTACGACCAGCAGAGGATC
	Reverse primer	GGCATCTGACAGTTCCTCCATG
Ccna1(Mouse)	Forward primer	GCTACTGAGGATGGAGCATCTG
	Reverse primer	CAGCTTCCAGAAGGCTCAGTTC
Ccne1(Mouse)	Forward primer	TGCCAAGGGAGAGAGACTCG
	Reverse primer	ATCCCAGGGCTGACTGCTAT
Cdc45(Mouse)	Forward primer	TGACCTTGAGGTTCCTGCCTAC
	Reverse primer	CCTCTTCCTGTTTCGCTCCACT
Cdk6(Mouse)	Forward primer	TGGATAAAGTTCCAGAGCCCG
	Reverse primer	CGGTTTCAGATCACGATGCAC
E2f1(Mouse)	Forward primer	GGATCTGGAGACTGACCATCAG
	Reverse primer	GGTTTCATAGCGTGACTTCTCCC
Tudor-SN (Rat)	Forward primer	GCTACGCAACCAGATGGGAAA
	Reverse primer	CTCGTCCTTGGGGAGTCTTGT
Gapdh (Rat)	Forward primer	CATCACTGCCACTCAGAAGACTG
	Reverse primer	ATGCCAGTGAGCTTCCCGTTCAG
Aurkb (Rat)	Forward primer	TTGCCCCAGAGAGTCCTACG
	Reverse primer	CAGTGGCACCCTTGTTCTCA
Ccna1(Rat)	Forward primer	AGGGAAATTGCAGCTTGTCG
	Reverse primer	CATCCTCAGTAGCTGTCGCT
Ccne1(Rat)	Forward primer	ACAAGACTGTGAAAAGCCAGGA
	Reverse primer	ATGCAGTCTTGGGGTACTCA
Cdc45(Rat)	Forward primer	GCCCTGTTTCAATGTGACCA
	Reverse primer	GCGCCACAGTTTATGAGGATG
Cdk6(Rat)	Forward primer	ACCTGGAGACCTTTGAGCAC
	Reverse primer	CCGGGTTCTGGAACTTTATCC
E2f1(Rat)	Forward primer	CCATCAGTACCTTGCTGGTAGC
	Reverse primer	CAAGAAGCGTTTGGTGGTCAG

Table S6

Table S6 Primer sequences of GST plasmids

Gene	Primer
GST-*Tudor-SN*-Forward primer	ATCTGGTTCCGCGTGGAATGGCCTCCGCGCAGAGCAGC
GST-*Tudor-SN*-Reverse primer	TCGAGTCGACCCGGGTTAGCGACTGTAGCCAAACTCA
GST-SN-Forward primer	CCGGCCTACTATGACTTATTCATCTCGAGATGAATAAGTCATAGTAGGTTTTTG
GST-SN-Reverse primer	AATTCAAAAACCTACTATGACTTATTCATCTCGAGATGAATAAGTCATAGTAGG
GST-TSN-Forward primer	ATCTGGTTCCGCGTGGAATGGCCTCCGCGCAGAGCAGC
GST-TSN-Reverse primer	AATTCAAAAACCTACTATGACTTATTCATCTCGAGATGAATAAGTCATAGTAGG
GST-*Yap*-Forward primer	ATCTGGTTCCGCGTGTTATGGATCCCGGGCAGC
GST-*Yap*-Reverse primer	TCGAGTCGACCCGGGCTATAACCATGTAAGAAAGCTTTCT
GST-TBD-Forward primer	ATCTGGTTCCGCGTGTTATGGATCCCGGGCAGC
GST-TBD-Reverse primer	TCGAGTCGACCCGGGTTTGGGCTCCGGCGGCTT
GST-WW-Forward primer	ATCTGGTTCCGCGTGTTATGTCCCACTCCCGAC
GST-WW-Reverse primer	TCGAGTCGACCCGGGTCTCTGGTTCATGGCAAAACGA
GST-AD-Forward primer	ATCTGGTTCCGCGTGTTATGATCAGTCAGAGTGCTCC
GST-AD-Reverse primer	TCGAGTCGACCCGGGCTATAACCATGTAAGAAAGCTTTCT
PcDNA3.1-HA-*Tudor-SN*-Forward primer	TTGGTACCGAGCTCGATGTACCCATACGATGTTCCAGATTACGCTATGGCCTCCGCGCAGAGCAGC
PcDNA3.1-HA-*Tudor-SN*-Reverse primer	GCTGGATATCTGCAGTTAGCGACTGTAGCCAAACTC
PcDNA3.1-Flag-*Yap*-Forward primer	TTGGTACCGAGCTCGATGGATTACAAGGATGACGACGATAAGATGGAGCCCGCGCAACAGCCG
PcDNA3.1-Flag-*Yap*-Reverse primer	GCTGGATATCTGCAGCTATAACCACGTGAGAAAGCTT
