# Supplementary material for: Tudor-SN promotes cardiomyocyte proliferation and neonatal heart regeneration through regulating the phosphorylation of YAP
Source: Cell Commun Signal. 2024 Jun 28;22:345. doi: 10.1186/s12964-024-01715-6 (PMC11212424; doi:10.1186/s12964-024-01715-6)

Supporting information

Additional file 2.

Uncut gel blot

Figure S1B

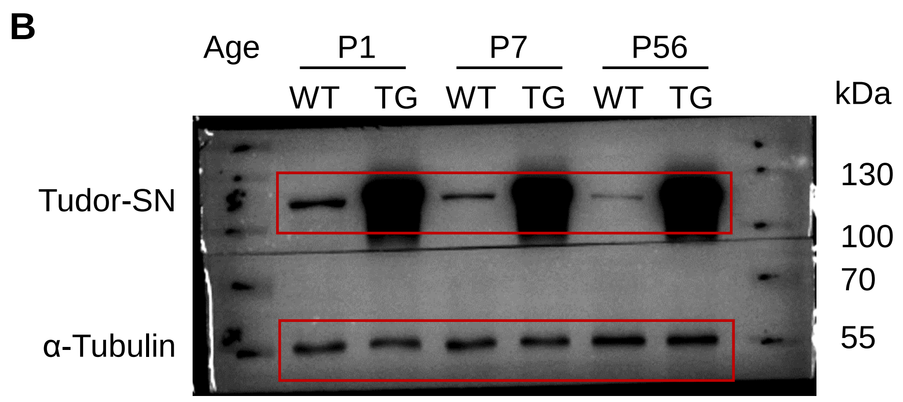

Figure S3D

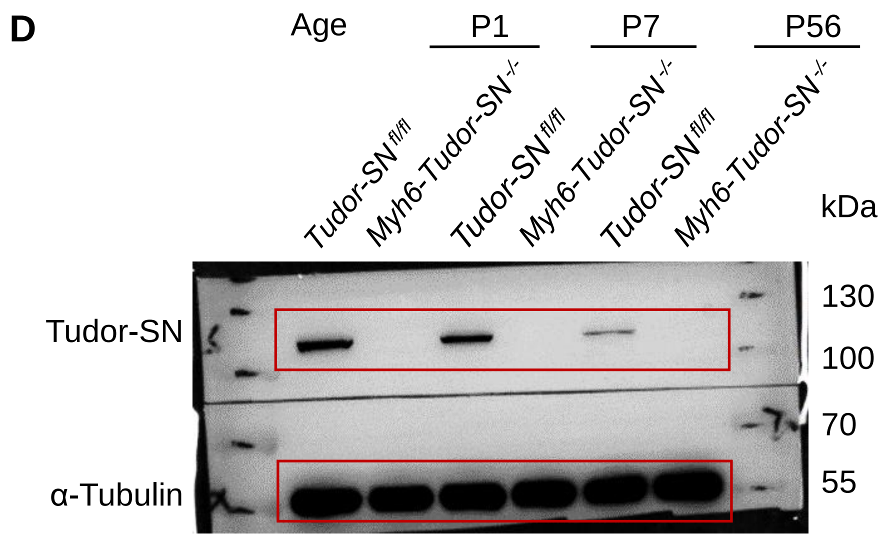

Figure 5 B-E

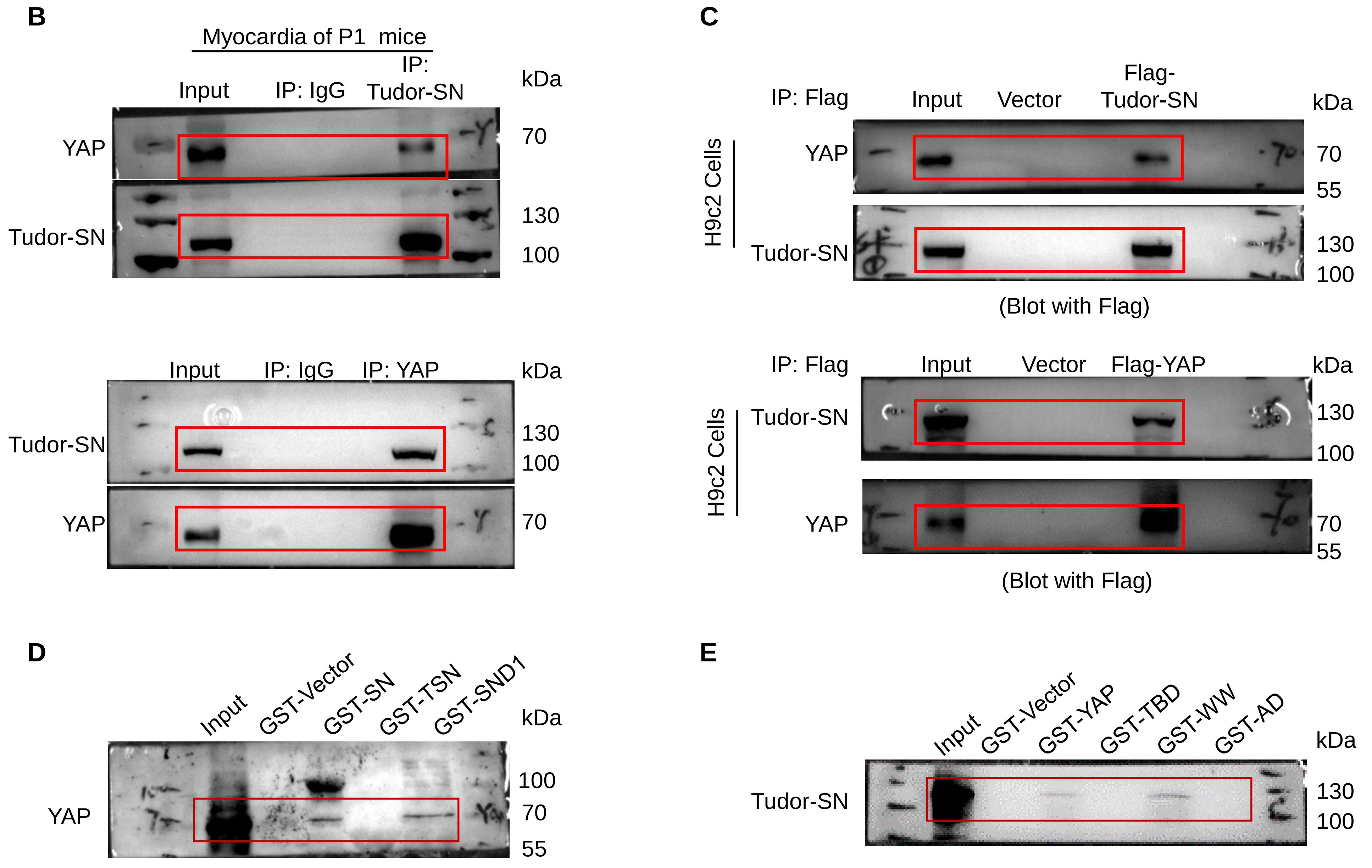
B
Myocardia of P1 mice
Input
IP: IgG
IP: Tudor-SN
kDa
YAP
Tudor-SN
70
130
100
Input
IP: IgG
IP: YAP
kDa
Tudor-SN
YAP
130
100
70
C
IP: Flag
Input
Vector
Flag-Tudor-SN
kDa
H9c2 Cells
YAP
Tudor-SN
70
55
130
100
(Blot with Flag)
IP: Flag
Input
Vector
Flag-YAP
kDa
H9c2 Cells
Tudor-SN
YAP
130
100
70
55
(Blot with Flag)
D
Input
GST-Vector
GST-SN
GST-TSN
GST-SND1
kDa
YAP
100
70
55
E
Input
GST-Vector
GST-YAP
GST-TBD
GST-WW
GST-AD
kDa
Tudor-SN
130
100

Figure 5 F-I

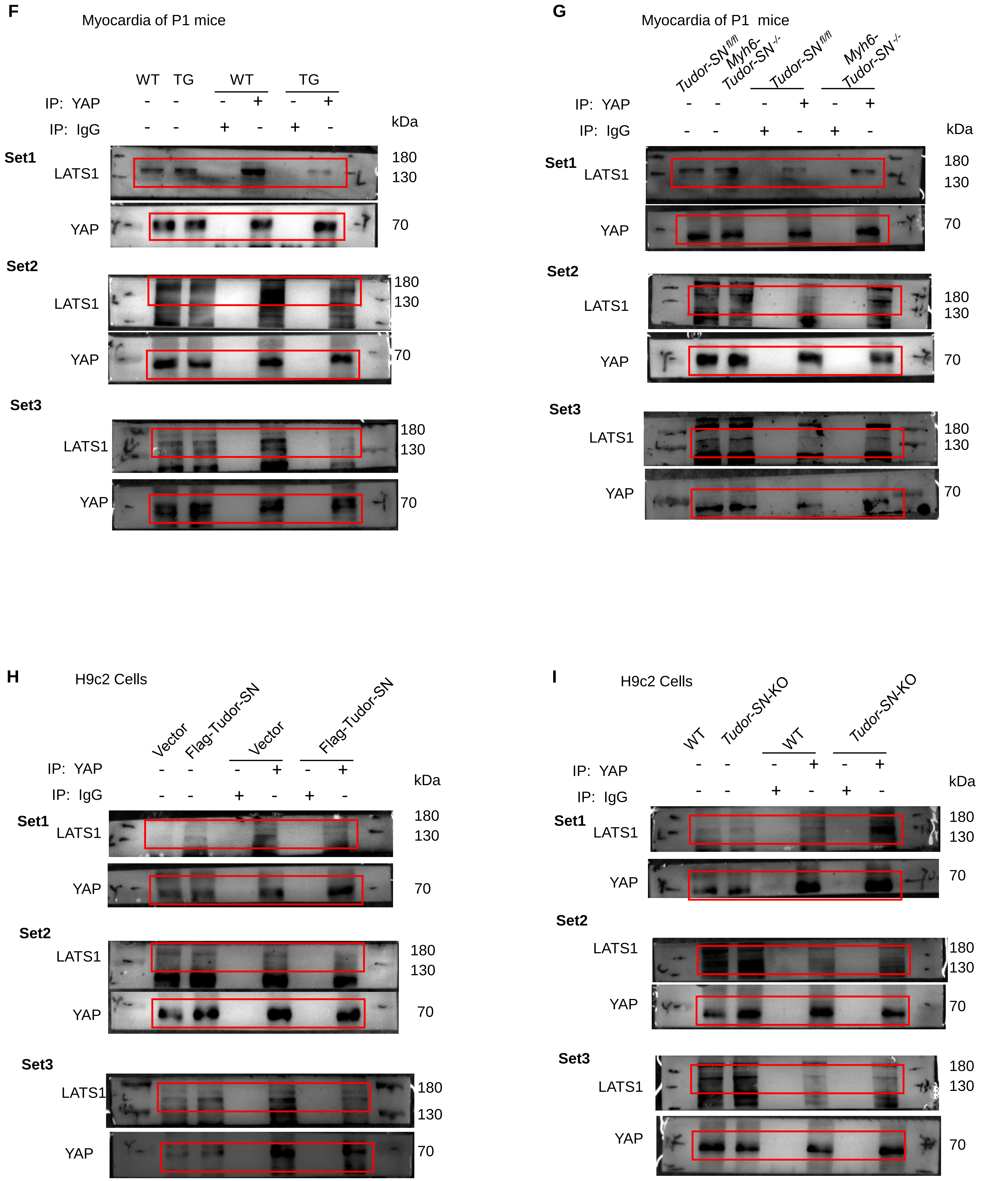

F
Myocardia of P1 mice
WT TG WT TG
IP: YAP - - - + - +
IP: IgG - - + - + -
kDa
Set1
LATS1
180
130
YAP
70
Set2
LATS1
180
130
YAP
70
Set3
LATS1
180
130
YAP
70
G
Myocardia of P1 mice
Tudor-SN fl/fl
Myh6-Tudor-SN -/-
Tudor-SN fl/fl
Myh6-Tudor-SN -/-
IP: YAP - - - + - +
IP: IgG - - + - + -
kDa
Set1
LATS1
180
130
YAP
70
Set2
LATS1
180
130
YAP
70
Set3
LATS1
180
130
YAP
70
H
H9c2 Cells
Vector
Flag-Tudor-SN
Vector
Flag-Tudor-SN
IP: YAP - - - + - +
IP: IgG - - + - + -
kDa
Set1
LATS1
180
130
YAP
70
Set2
LATS1
180
130
YAP
70
Set3
LATS1
180
130
YAP
70
I
H9c2 Cells
WT
Tudor-SN-KO
WT
Tudor-SN-KO
IP: YAP - - - + - +
IP: IgG - - + - + -
kDa
Set1
LATS1
180
130
YAP
70
Set2
LATS1
180
130
YAP
70
Set3
LATS1
180
130
YAP
70

Figure 6 A-D

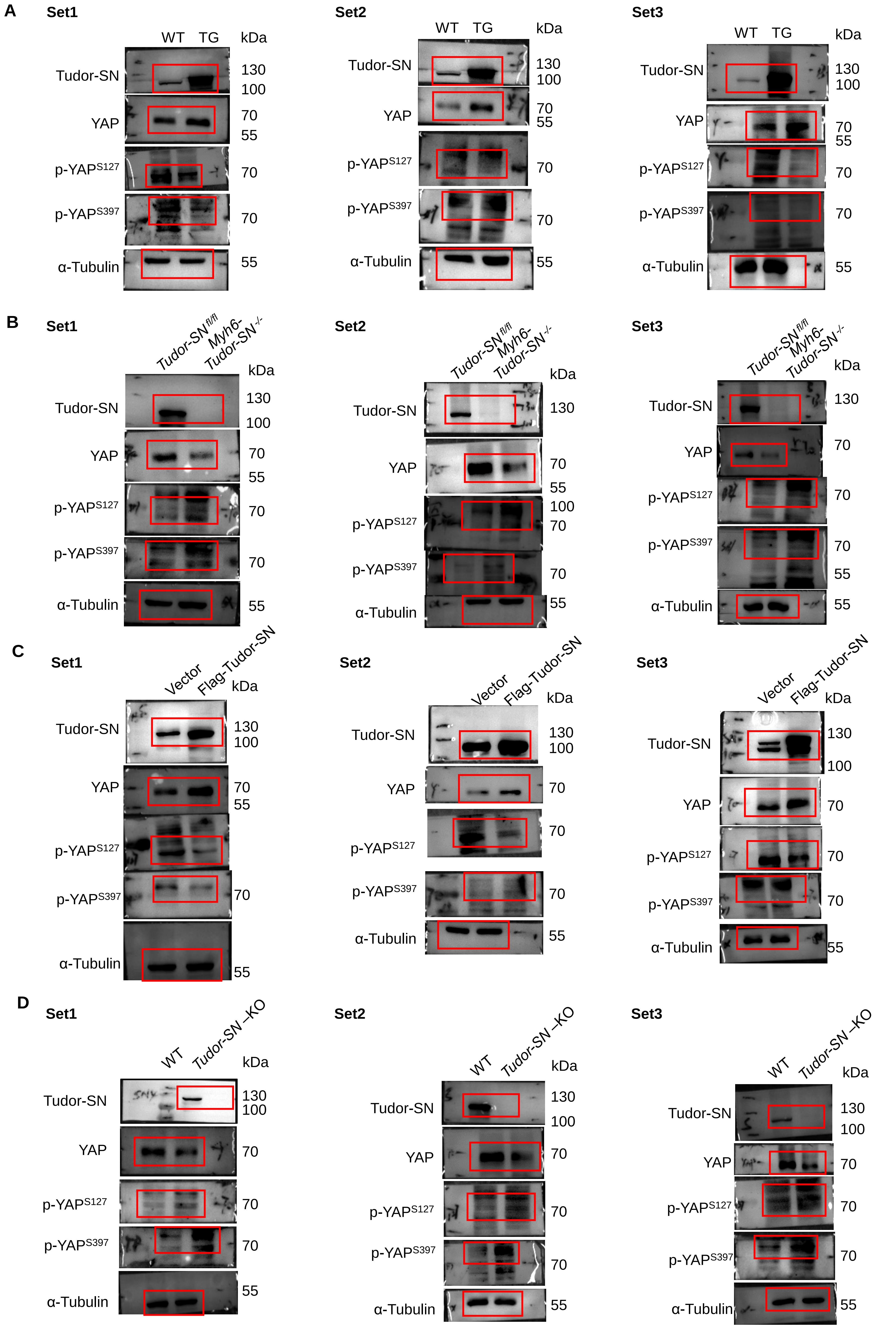
A
Set1
WT
TG
kDa
Tudor-SN
YAP
p-YAP^{S127}
p-YAP^{S397}
α-Tubulin
130
100
70
55
Set2
Set3
B
Tudor-SN$^{fl/fl}$
Myh6-Tudor-SN$^{-/-}$
C
Vector
Flag-Tudor-SN
D
Tudor-SN –KO

Figure 6 E-H

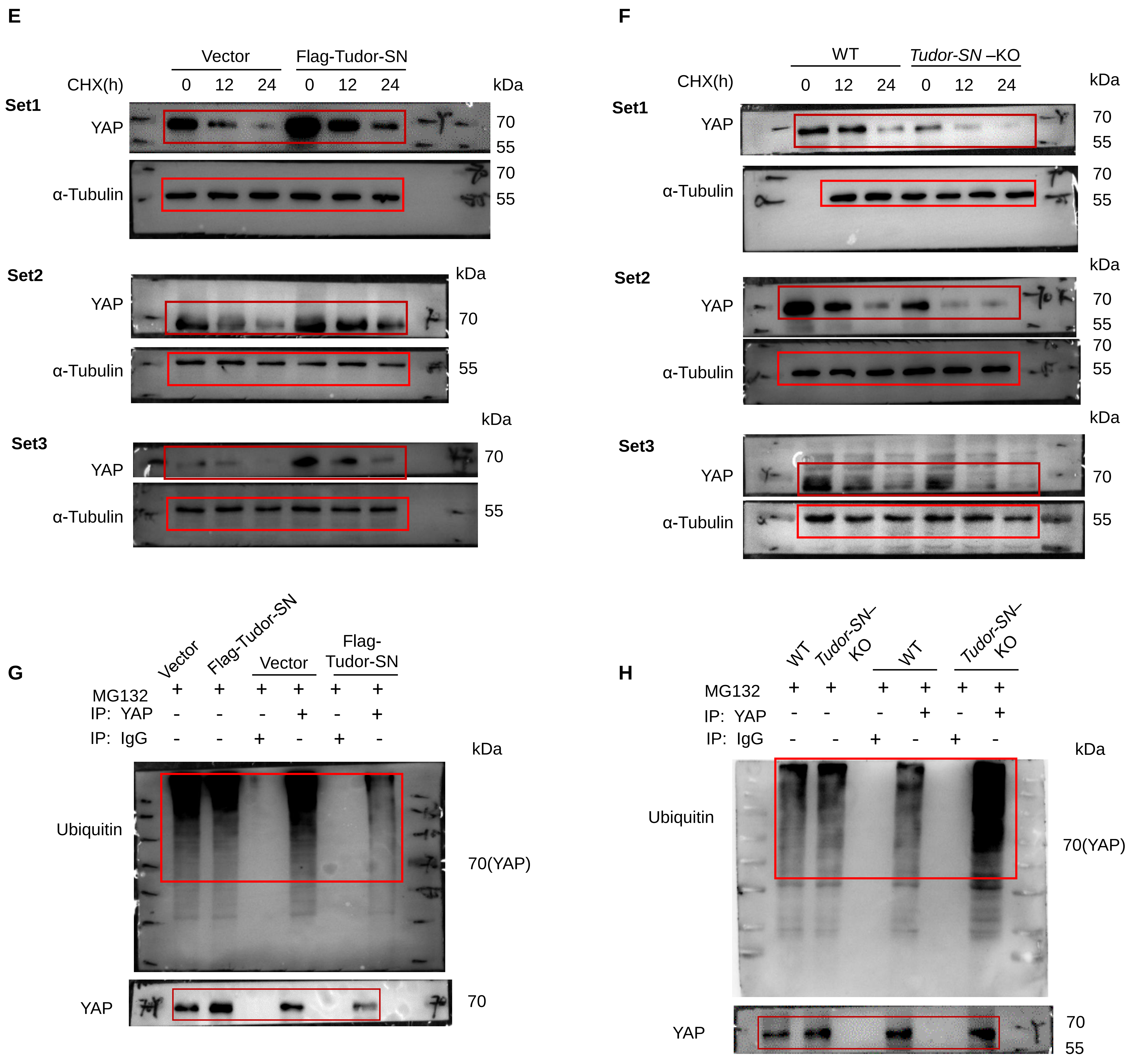
E
Vector
Flag-Tudor-SN
CHX(h)
0 12 24 0 12 24
kDa
Set1
YAP
70
55
α-Tubulin
70
55
Set2
kDa
YAP
70
α-Tubulin
55
Set3
kDa
YAP
70
α-Tubulin
55
F
WT
Tudor-SN –KO
CHX(h)
0 12 24 0 12 24
kDa
Set1
YAP
70
55
α-Tubulin
70
55
Set2
kDa
YAP
70
55
α-Tubulin
70
55
Set3
kDa
YAP
70
α-Tubulin
55
G
Vector
Flag-Tudor-SN
Vector
Flag-Tudor-SN
MG132 + + + + + +
IP: YAP - - - + - +
IP: IgG - - + - + -
kDa
Ubiquitin
70(YAP)
YAP
70
H
WT
Tudor-SN–KO
WT
Tudor-SN–KO
MG132 + + + + + +
IP: YAP - - - + - +
IP: IgG - - + - + -
kDa
Ubiquitin
70(YAP)
YAP
70
55

Figure S9 A-D

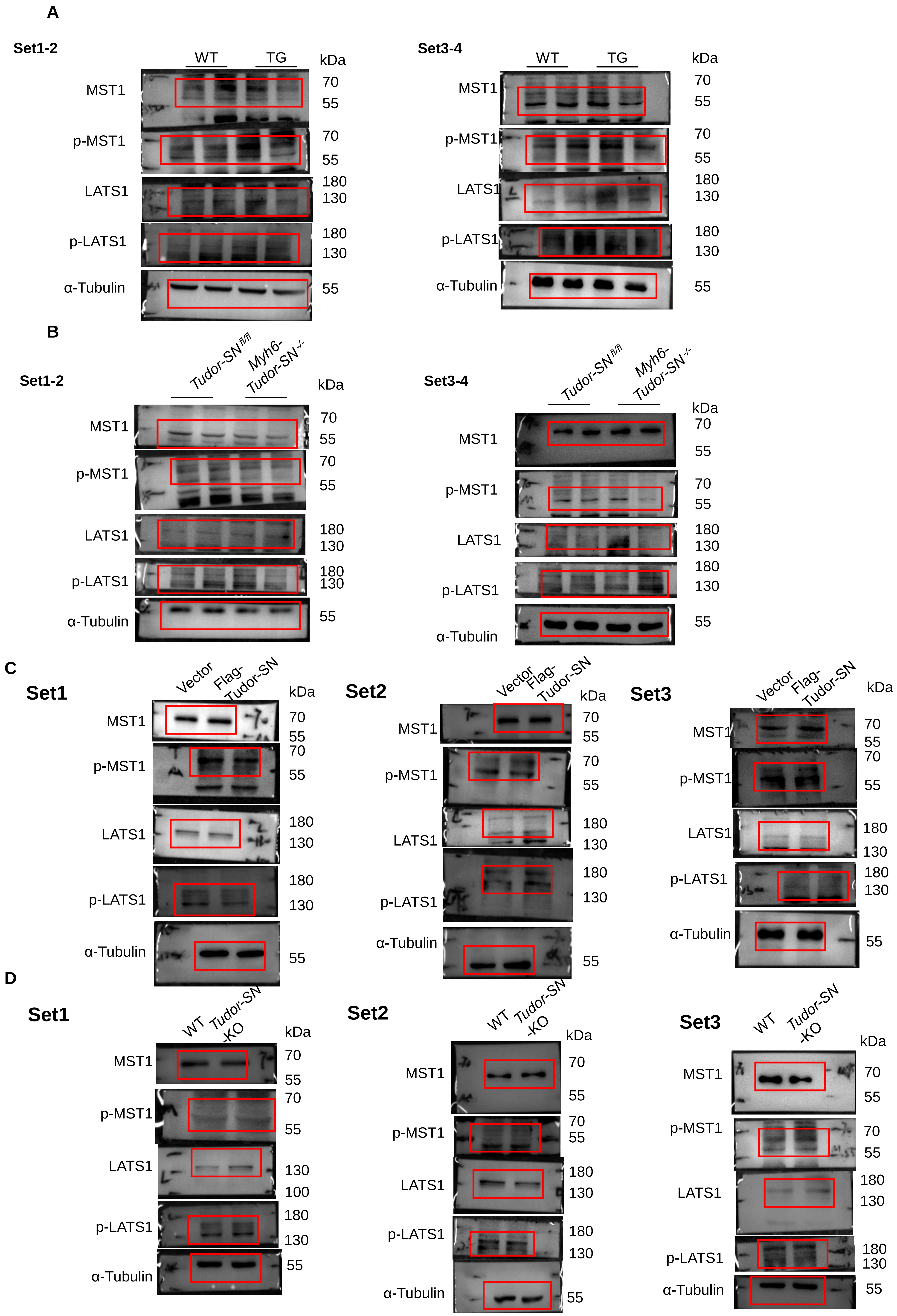
A
Set1-2
WT
TG
kDa
MST1
70
55
p-MST1
70
55
LATS1
180
130
p-LATS1
180
130
α-Tubulin
55
Set3-4
WT
TG
kDa
MST1
70
55
p-MST1
70
55
LATS1
180
130
p-LATS1
180
130
α-Tubulin
55
B
Set1-2
Tudor-SN$^{fl/fl}$
Myh6-Tudor-SN$^{-/-}$
kDa
MST1
70
55
p-MST1
70
55
LATS1
180
130
p-LATS1
180
130
α-Tubulin
55
Set3-4
Tudor-SN$^{fl/fl}$
Myh6-Tudor-SN$^{-/-}$
kDa
MST1
70
55
p-MST1
70
55
LATS1
180
130
p-LATS1
180
130
α-Tubulin
55
C
Set1
Set2
Set3
Vector
Flag-Tudor-SN
kDa
MST1
70
55
p-MST1
70
55
LATS1
180
130
p-LATS1
180
130
α-Tubulin
55
D
Set1
Set2
Set3
WT
Tudor-SN -KO
kDa
MST1
70
55
p-MST1
70
55
LATS1
130
100
180
130
p-LATS1
180
130
α-Tubulin
55

Figure 7 A-B

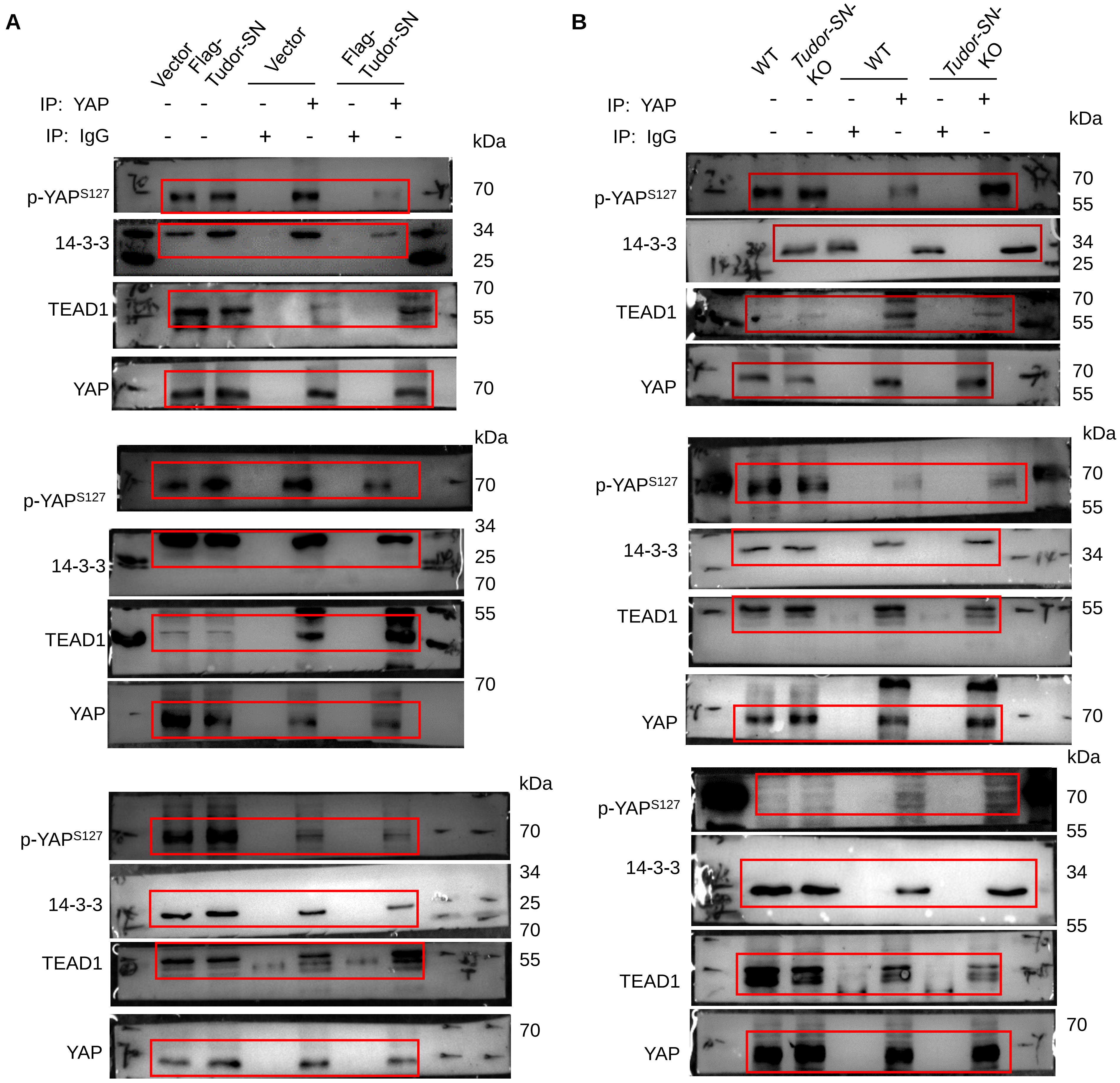
A
Vector
Flag-Tudor-SN
Vector
Flag-Tudor-SN
IP: YAP
IP: IgG
kDa
p-YAPS127
14-3-3
TEAD1
YAP
70
34
25
55
B
WT
Tudor-SN-KO
WT
Tudor-SN-KO
IP: YAP
IP: IgG
kDa
p-YAPS127
14-3-3
TEAD1
YAP
70
55
34
25

Figure 7 E-H

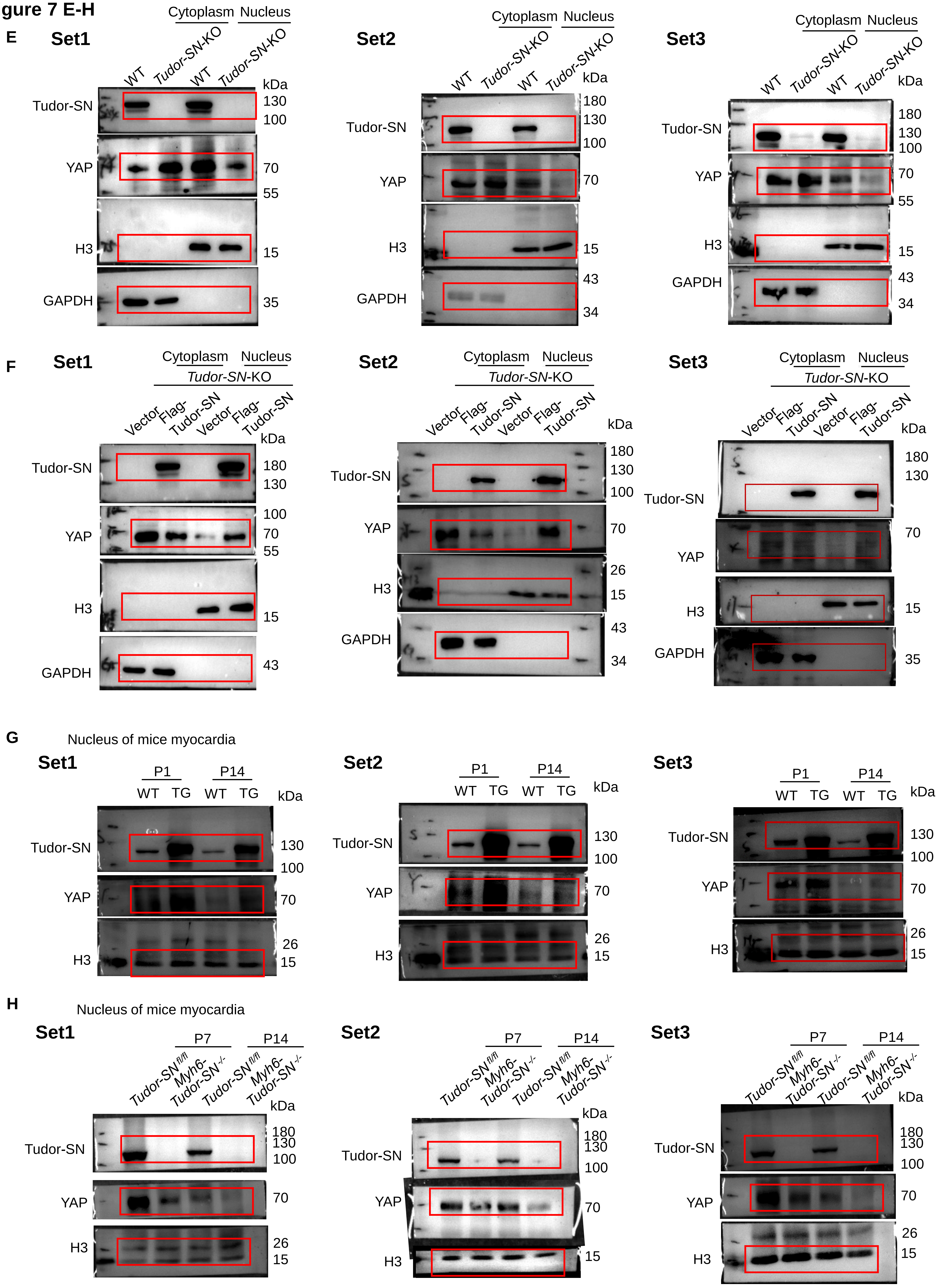
E
Set1
Set2
Set3
Cytoplasm
Nucleus
WT
Tudor-SN-KO
kDa
Tudor-SN
YAP
H3
GAPDH
180
130
100
70
55
15
43
35
34
F
Tudor-SN-KO
Vector
Flag-Tudor-SN
26
G
Nucleus of mice myocardia
P1
P14
TG
H
P7
Tudor-SN fl/fl
Myh6-Tudor-SN -/-

Figure S7 A

A

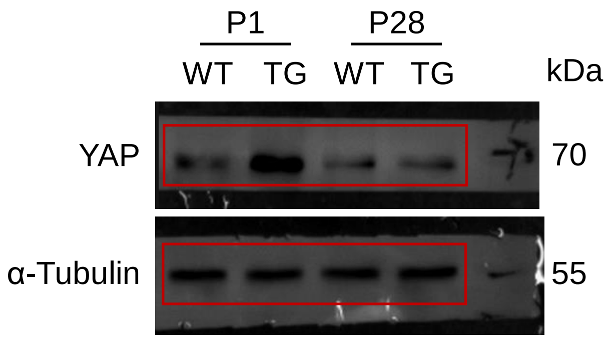

Supplement: Supplementary file 2 — Additional file 2. Uncut gel blot. [file 12964_2024_1715_MOESM2_ESM.pdf]
